# The phonological store of working memory: A critique and an alternative, perceptual-motor, approach to verbal short-term memory

**DOI:** 10.1177/17470218241257885

**Published:** 2024-07-26

**Authors:** Robert W Hughes

**Affiliations:** Department of Psychology, Royal Holloway, University of London, Egham, UK

**Keywords:** Phonological store, verbal serial short-term memory, working memory, serial recall, perceptual-motor approach

## Abstract

A key quality of a good theory is its fruitfulness, one measure of which might be the degree to which it compels researchers to test it, refine it, or offer alternative explanations of the same empirical data. Perhaps the most fruitful element of Baddeley and Hitch’s (1974) Working Memory framework has been the concept of a short-term *phonological store*, a discrete cognitive module dedicated to the passive storage of verbal material that is architecturally fractionated from perceptual, language, and articulatory systems. This review discusses how the phonological store construct has served as the main theoretical springboard for an alternative *perceptual-motor* approach in which serial-recall performance reflects the opportunistic co-opting of the articulatory-planning system and, when auditory material is involved, the products of obligatory auditory perceptual organisation. It is argued that this approach, which rejects the need to posit a distinct short-term store, provides a better account of the two putative empirical hallmarks of the phonological store—the phonological similarity effect and the irrelevant speech effect—and that it shows promise too in being able to account for nonword repetition and word-form learning, the supposed evolved function of the phonological store. The neuropsychological literature cited as strong additional support for the phonological store concept is also scrutinised through the lens of the perceptual-motor approach for the first time and a tentative articulatory-planning deficit hypothesis for the “short-term memory” patient profile is advanced. Finally, the relation of the perceptual-motor approach to other “emergent-property” accounts of short-term memory is briefly considered.

The most researched, most fully specified, and, arguably, most influential component of Baddeley and Hitch’s (1974) Working Memory model is the phonological loop, a discrete system specialised for the short-term retention of verbal or verbalisable input. The core structure within the phonological loop, in turn, is the phonological store, a passive short-term store that holds representations of verbal items in phonological form for around 2 s before they are lost to decay (Baddeley, 1986, 2007). The decaying item representations can be revivified via an active articulatory control process that supports articulatory rehearsal. The articulatory control process must also be engaged to convert visually presented input into phonological form (grapheme-to-phoneme conversion) while auditory–verbal input gains obligatory access to the store as such input is already in phonological form. Logically, the fact that the store can receive its input via acoustic analysis (for auditory–verbal material) in the absence of articulation, but also via articulation (for visual–verbal material) in the absence of any acoustic input (the articulation need not be audible), indicates that the representations therein indeed lie at a central, post-categorical (i.e., “phonological”) level. If we add to this the basic claim that the store is specialised for verbal information, whether this is derived from acoustic analysis or articulatory processing, then the aptness of the term *phonological* store is brought further into relief: Its units of currency are post-categorical representations of the constituent sounds of a language (Chomsky & Halle, 1968). The phonological store interfaces with long-term memory (LTM), specifically, with long-term knowledge of phonological word forms (e.g., Gathercole, 1995), and with acoustic-perceptual analysis, particularly of speech. Nevertheless, the store is considered to be a distinct cognitive module, architecturally separate from general perceptual, language, and articulatory (or, more generally, motor) systems (Baddeley, 2012).

For 50 years, the phonological loop construct has been hugely fruitful in terms of catalysing a large and rich body of research on verbal serial short-term memory (STM) and in galvanising a healthy competition between different theoretical views on the subject within cognitive psychology (e.g., Baddeley & Larsen, 2007; Cowan, 1999; Jones et al., 2004; Larsen & Baddeley, 2003; Nairne, 2002; Neath & Nairne, 1995), developmental psychology (e.g., Gathercole, 2006; Melby-Lervåg et al., 2012), cognitive neuropsychology (e.g., Buchsbaum & D’Esposito, 2019; Caplan et al., 2012; Vallar & Papagno, 2002), and cognitive neuroscience (Buchsbaum & D’Esposito, 2008; Shallice & Papagno, 2019). From its inception, its main strength has been the elegant way in which the interplay of the components that make up its relatively simple architecture—a passive, decay-prone, phonological store supported by an articulatory control process—appears to provide a good account of a relatively large number of key verbal serial-recall phenomena.

In the current review, however, I will seek first to make the case that the two empirical signatures of the phonological store—the phonological similarity effect and the irrelevant speech effect—are better explained by recourse to articulatory-planning processes (regardless of presentation modality) and acoustic-perceptual organisation processes (when auditory input is involved), without assuming the existence of a specialised passive phonological store. Having discussed the perceptual-motor approach in some further detail, I then use the framework to reevaluate the neuropsychological literature on the “short-term memory patient” in which there is an apparent selective deficit of the phonological store and which has, therefore, been taken as strong additional support for the phonological store construct (e.g., Vallar, 2006). A part of this section will also involve a brief consideration of brain imaging research that purports to have isolated the “neural correlate” of the phonological store (Baddeley, 2012). Next, I will evaluate some of the key evidence that has been taken to support the view that the evolved function of the phonological store is not verbal short-term term retention per se, but the long-term verbal sequence learning that such short-term retention affords (Baddeley et al., 1998; Gathercole, 2006). Some recent research from my lab will be reviewed suggesting that articulatory planning plays a much more prominent role in verbal sequence learning than previously thought (e.g., Hughes et al., 2024; Sjöblom & Hughes, 2020). Finally, I will discuss briefly how the perceptual-motor view relates conceptually to other “emergent-property” accounts of verbal serial STM.

## The phonological similarity effect

The main empirical signature of the passive phonological store is the *phonological similarity effect*: A list such as “man, mad, cap. . .” or “B, G, D. . .” is more poorly recalled in order than a list such as “pen, day, sup. . .” or “B, Q, F. . .” (Baddeley, 1966; Baddeley et al., 1984; Conrad, 1964; Conrad & Hull, 1964; Hintzman, 1967; Wickelgren, 1965). On the phonological loop model, this effect reflects the greater confusability of representations of similar items during retrieval from the store. It is important to stress that the phonological similarity effect, however, does not in and of itself provide support for the passive phonological store construct. Indeed, the original explanation of the effect within the Working Memory framework was that it reflected articulatory, not phonological, processes (Baddeley & Hitch, 1974; Vallar & Baddeley, 1984; for precursors of this view, see Hintzman, 1965, 1967; Levy & Murdock, 1968; D. J. Murray, 1968; Wickelgren, 1965, 1966).

One of the key observations that instigated the need to propose a passive phonological store in addition to articulatory processes within the verbal component of the Working Memory model was the particular way in which the phonological similarity effect was found to interact with two other variables: Presentation modality—whether the to-be-remembered items are presented visually or auditorily—and articulatory suppression (Baddeley et al., 1984; see also Salamé & Baddeley, 1982, and the following section). Articulatory suppression refers to the requirement for a participant to cyclically utter (in subvocal, whispered, or vocalised manner) an irrelevant word or sequence (e.g., “the, the, the. . .” or “x, y, z, x. . .”) during the presentation of the to-be-remembered items, during a retention interval (if one is included) between the last to-be-remembered item and a recall cue, or both (e.g., Baddeley, 1986; Jones et al., 2004; D. J. Murray, 1968). It was observed that with visual presentation, the phonological similarity effect disappears under articulatory suppression (Baddeley et al., 1984; D. J. Murray, 1968; Peterson & Johnson, 1971; Wilding & Mohindra, 1980). This was consistent with the original articulatory account: If the effect has an articulatory basis, then impeding articulation should eliminate it. However, critically, it was found that with auditory presentation, the phonological similarity effect survives articulatory suppression (Baddeley et al., 1984; Levy, 1971; D. J. Murray, 1968; Peterson & Johnson, 1971). The articulatory account was thus rejected (Baddeley et al., 1984; Vallar & Baddeley, 1984) and instead it was inferred that there must be a passive phonological store that receives input automatically and without the intervention of active articulatory processes so long as the input is *auditory*–verbal and hence already in phonological form. The extent to which the concept of a phonological store rests on this three-way interaction is difficult to overestimate. Baddeley (1986), for example, recalls how:

(T)he particular pattern of results obtained was crucial to separating the two components of the articulatory loop, the phonological store and the articulatory control process. Had the results not worked out in this way, it would have been necessary to modify the model quite seriously. (p. 257)

Subsequent studies have shown that the critical three-way interaction does indeed not work out in the way that supports the postulation of a passive phonological store. Jones et al. (2004) replicated the finding that suppression eliminates the phonological similarity effect with visual lists. They also replicated the finding that the phonological similarity effect survives under suppression with auditory lists. However, critically, this survival was observed primarily for the last few items in the list, that is, at recency (see also Sjöblom & Hughes, 2020). It turns out that a much earlier study by Murray (1968) had observed the same pattern in the context of a probed order task: “Recall of the *final items* of nonarticulated auditory lists is affected by AC [acoustic confusability] . . .the effects of AC were also marked on early items *but only when rehearsal was permitted*” (p. 683; emphasis added). Jones et al. (2004) argued, therefore, that the residual similarity effect is a product of the *modality effect* (or *auditory recency*), the enhanced recall of the last one or two items in an auditorily presented compared with visually presented list (Crowder, 1978; Nicholls & Jones, 2002), a phenomenon that is deemed to be “peripheral to the working memory system” (Baddeley, 1986, p. 95; see also Hurlstone et al., 2014). More precisely, the residual effect is a product of the fact that auditory recency shows an *acoustic* similarity effect (e.g., Crowder, 1971, 1978; Darwin & Baddeley, 1974; Watkins et al., 1974). That is, auditory recency is much reduced, if not absent, for similar items such as “B, C, G. . .”—which are, when presented auditorily, acoustically, and not just phonologically similar to one another—compared with “J, R, Q. . ..” It is this acoustic similarity effect at recency that survives articulatory suppression. Accordingly, when auditory recency is eliminated with the addition of a suffix—a redundant spoken item appended to the end of the auditory list—this acoustic similarity effect under suppression also disappears (Jones et al., 2004). Moreover, it can be made to appear again if an irrelevant acoustic sequence is presented such as to perceptually “capture” the suffix into a stream separate from that formed by the to-be-remembered list (Maidment & Macken, 2012). Thus, in sum, the residual “phonological” similarity effect can be made to come and go by manipulating acoustic factors that modulate the accessibility of items at the end-boundary of the list (as well as those earlier in the list with very short lists; Jones et al., 2006; see also below).

In response, Baddeley (2007) noted that “[t]here is no doubt that the effect identified by Jones and colleagues offers a challenge to the existing hypothesis. . .” (p. 56). It was then suggested, however, that the fact that the survival of the phonological similarity effect was observed primarily at recency in the data of Jones et al. (2004) may have been due to the phonological store being overloaded—given the addition to a 7-item list of letters of a requirement for articulatory suppression—and participants therefore abandoning the use of the phonological store in favour of some other, unspecified, recall strategy (Baddeley, 2007; Baddeley & Larsen, 2007; see also Salamé & Baddeley, 1986). It is worth noting first that this suggestion, in claiming that the phonological store was not used, implicitly acknowledges that the “phonological” similarity effect that Jones et al. (2004) observed mainly at recency could indeed not, therefore, have been a phonological similarity effect, in line with the view that it was an acoustic similarity effect. That is, the suggestion is that, in addition to a non-phonological similarity effect at recency (as observed by Jones et al., 2004), the “true” phonological similarity effect does survive suppression with auditory lists more generally; it is just that Jones et al. (2004) “missed” this effect due to their participants being overloaded and abandoning the phonological store.

Given its critical importance for potentially providing a reprieve for the phonological store concept, it is worth taking a closer look at the store-abandonment hypothesis (see also Jones et al., 2007). Consistent with the notion that participants may indeed have abandoned the phonological store in the critical conditions of Jones et al.’s (2004) study, Baddeley and Larsen (2007) reported an experiment in which the survival of the phonological similarity effect under suppression with auditory lists was evident throughout the list when 6-item lists were used instead of 7-item lists. However, a curious feature of the Baddeley and Larsen (2007) experiment in the context of the issue in question is that it included a 10-s retention interval during which participants were to continue suppressing (in addition, and also unusually, the experiment did not have a no-suppression control condition). It is far from clear, therefore, why representations in the phonological store would not have long been lost to decay, leading to the prediction of no phonological similarity effect, contrary to the data. Indeed, based on the results of an experiment in which the rate of presentation of items in an auditory list was varied (1 item per 3 s with 1 item per 0.5 s), Baddeley and Lewis (1984) suggested that when “rehearsal is prevented by suppression, under conditions of slow presentation, the memory trace will have time to decay before recall is required” (p. 404). Moreover, Fournet et al. (2003) showed that the phonological similarity effect (with visual lists) is present with a 2-s filled retention interval but disappears after an 8-s filled retention interval.

If, however, we take the result of Baddeley and Larsen (2007) at face value, the pattern of data is, in any case, in line with a study by Jones et al. (2006), which also used short lists (5 items) (but note that they did not include a retention interval but did include a no-suppression control); they also observed a phonological similarity effect throughout an auditory list under suppression using such short lists. The key difference from the Baddeley and Larsen (2007) experiment, however, is that Jones et al. (2006) took the further step of examining whether that throughout-list effect could again be understood by recourse to acoustic-based perceptual organisation rather than phonological storage. In their Experiment 2, they added a suffix (as in Jones et al., 2004) but also a prefix, to reduce the perceptual accessibility of to-be-remembered items at the list-initial boundary as well as list-end boundary. Under these conditions, the phonological similarity effect again disappeared throughout the list. Reinforcing the acoustic basis of the effect, in Jones et al.’s (2006) Experiment 3, the phonological similarity effect was reinstated again simply by making the voice of the (phonologically unchanged) prefix and suffix acoustically different from that delivering the to-be-remembered items. Moreover, the absence of the similarity effect when the redundant items were in the same voice as the list (Experiment 2) and the re-emergence of the effect when the redundant items were in a different voice from the list (Experiment 3) was observed even though the overall level of performance in the two experiments was virtually identical. It is not plausible, therefore, to suggest that participants used the phonological store in the experiment that showed a phonological similarity effect (Experiment 3), but abandoned it in the experiment in which it was absent (Experiment 2) (for further evidence against the store-abandonment account, see Maidment & Macken, 2012).

It has been argued, therefore, that the “phonological similarity effect” is a misnomer (Jones et al., 2004, 2006, 2007; Maidment & Macken, 2012; Sjöblom & Hughes, 2020). The effect observed when participants are free to engage in articulatory rehearsal, with both visual and auditory lists, is primarily a product of that articulatory process itself (as suggested in the original Baddeley & Hitch, 1974, formulation of the Working Memory model; see also Ellis, 1980; A. W. Hintzman, 1965, 1967; Levy & Murdock, 1968; Wickelgren, 1965). Specifically, the articulatory similarity effect results from the involuntary transposition of speech elements during articulatory planning (so-called spoonerisms or “slips of the tongue”; e.g., saying “overinstated flate” instead of “overinflated state”; Goldstein, 1968; MacKay, 1970). Indeed, the pattern of errors found in the serial recall of “phonologically” similar items is identical to that found when lists are read (without appreciable memory load) or when found in spontaneous speech (Acheson & MacDonald, 2009; Ellis, 1980; MacKay, 1970; Page et al., 2007; Shattuck-Hufnagel & Klatt, 1979). Thus, in the above example, for instance, the consonant clusters at the onset of each stressed syllable in the intended phrase (“fl” in “flated” and “st” in “state”) are prone to switching places within the articulatory plan because each is followed by a phonologically similar (indeed identical) coda (i.e., “. . ate” in each case), just as the consonants in the letter-names B (“bee”) and D (“dee”) are prone to being transposed when presented within the phonologically similar serial-recall list “B, D, P. . .” due to the shared “ee” vowel sound, resulting in relatively frequent transposition errors such as “D, B, P. . .” (Henson, 1998; Page & Norris, 2009). In other words, the phonologically similar serial recall list is the ultimate “tongue twister” (Acheson & MacDonald, 2009a; Page et al., 2007).

It has been argued thus far that when participants are free to engage in articulatory planning (i.e., under no-suppression conditions), the “phonological” similarity effect—regardless of presentation modality—is a product of that speech planning process. If the formation of the motor programme is prevented through articulatory suppression, however, necessarily there will be no opportunity to make errors in planning and (re)producing the list and hence no articulatory similarity effect. However, when presentation is auditory, as discussed above, an acoustic similarity effect can also arise (at auditory recency with relatively long lists but also throughout the list with very short lists; Jones et al., 2006). This acoustic similarity effect is typically obscured or at least diluted in no-suppression conditions—due to the articulatory similarity effect that occurs under these conditions regardless of presentation modality—but the acoustic similarity effect comes to the fore when that articulatory similarity effect is dampened or abolished by articulatory suppression. This is because the acoustic similarity effect, unlike the articulatory similarity effect, is a product of automatic, pre-attentive, auditory perceptual-organisation processes that operate independently of the articulatory system (cf. Bregman, 1990). Such auditory perceptual organisation refers to the Gestalt processes by which the undifferentiated mixture of inputs received by the ears is partitioned into distinct perceptual objects or streams corresponding to the various distinct environmental events that contributed to that mixture (e.g., Koffka, 1935).

From this standpoint, acoustic similarity modulates serial recall performance by affecting the degree to which automatic auditory perceptual organisation yields information about order. It is well established that the perception of temporal order in an auditory sequence (verbal or otherwise) is a non-monotonic function of the acoustic similarity between its constituent elements and hence the degree to which the elements are fused into a single auditory object: When the elements are relatively acoustically similar to one another, order perception is relatively poor, despite the fact that such elements are most likely to perceived as belonging to a single coherent auditory object. When they are more distinct but nonetheless retain a common ground and hence still integrated into a single object (e.g., different items spoken in a common voice), this is when order perception is particularly strong. Finally, when the successive elements are so distinct from one another such that they fail to cohere into the same perceptual object (e.g., different items spoken in different voices), order perception is poor again (Bregman & Campbell, 1971; Hughes et al., 2009, 2011, 2016; Jones et al., 1999; Jones & Macken, 1995a; Lackner & Goldstein, 1974; Warren et al., 1969). This of course makes functional sense: There would, typically, be little functional utility to tracking the order of successive acoustic elements emanating from different environmental events; rather, it is the order of elements within a given auditory object (e.g., a particular talker) that is potentially important (Bregman, 1990). Thus, the fact that the modality effect is larger with “phonologically” dissimilar sequences can be understood in terms of the notion that the elements in a “phonologically” similar sequence are too *acoustically* similar to yield strong order cues.

In sum, detailed scrutiny of the way in which phonological similarity interacts with articulatory suppression and presentation modality indicates that the “phonological” similarity effect is not indicative of the existence of a passive post-categorical phonological short-term store that is, by definition, independent of articulatory and acoustic-perceptual processes (cf. Baddeley, 2007; Baddeley et al., 1984). The effect is primarily (and purely so with visual–verbal lists) a product of the opportunistic use of an error-prone articulatory-planning process (cf. Ellis, 1980), co-opted in support of the reproduction of a verbal list (see “A perceptual-motor approach” section below). In addition, an acoustic similarity effect can also masquerade as a phonological similarity effect with auditory lists, particularly when articulatory planning is impeded (Jones et al., 2004, 2006; Maidment & Macken, 2012; Sjöblom & Hughes, 2020).

## The irrelevant speech effect

The second key phenomenon thought to reflect and hence provide support for the existence of a passive phonological store is the disruption of verbal serial recall by irrelevant speech (e.g., Colle & Welsh, 1976; Hughes et al., 2007; Jones et al., 1992; LeCompte, 1996; Neath, 2000; Röer et al., 2015; Salamé & Baddeley, 1982, 1986). Importantly, this effect occurs even if the memoranda are presented visually—indeed the vast majority of studies of the phenomenon have involved visual–verbal serial recall—and regardless of whether the speech coincides with the presentation of the memoranda or is confined to a retention interval (Miles et al., 1991). These aspects of the effect indicate that the disruption is not due to some sort of peripheral (i.e., sensory) masking problem.

The irrelevant speech effect played an important role in establishing two founding principles of the phonological store construct (e.g., Baddeley, 1986): That it is a *phonological* store—and not, for example, an acoustic or semantic one—and that it is a *store*, that is, an entity into which (a certain type of) input gains obligatory entry independently of any other cognitive or motor process (e.g., articulation) and whose sole purpose is to “hold” the information temporarily. The first of these principles—that the store is phonological—seemed to enjoy support from a number of convergent findings: First, it appeared at one time that the irrelevant speech did indeed need to be speech—and hence be phonological in form—to disrupt verbal serial recall, with bursts of pink or white noise, for example, having very little if any effect compared with quiet (Salamé & Baddeley, 1982). Second, higher-order characteristics of speech such as meaning do not play any role in the effect. For example, speech in a language the participant does not understand is as disruptive as speech in a language they do understand (Colle & Welsh, 1976; Salamé & Baddeley, 1982). Third, it was reported that irrelevant speech tokens phonologically similar to the to-be-remembered items produced greater disruption than speech tokens that were phonologically dissimilar. Specifically, using the to-be-remembered digits 1–9, Salamé and Baddeley (1982, Experiment 5) found that presenting the same set of digits (in a different order from the to-be-remembered digits) as well as spoken tokens made up of the same phonemes as the digits but with their onset phonemes rearranged (e.g., “*sore*” and “ *fix*” as opposed to “*four*” and “*six*”) was more disruptive than a sequence of phonologically dissimilar words. Thus, just as to-be-remembered items that are phonologically similar to one another interfere with each other and are hence relatively poorly recalled, “it is the *degree of phonological similarity* between the irrelevant material and the memory items that underlies the irrelevant speech effect” (Gathercole & Baddeley, 1993, p. 13). Thus, on the phonological loop model, the fact that the speech is task-irrelevant and to be ignored—that is, not actively processed—indicates that there must be a store that automatically registers and temporarily holds phonological input.

A third finding from the study of the irrelevant speech effect that reinforced the notion of a passive phonological store separate from active articulatory processes came in the form of a three-way interaction between irrelevant speech, articulatory suppression, and the modality of the memoranda, mirroring the interaction found between these latter two variables and phonological similarity (see previous section): It had been observed that articulatory suppression eliminated the irrelevant speech effect with visual lists (e.g., Miles et al., 1991; Salamé & Baddeley, 1982; see also Hanley, 1997; Jones et al., 2004; Klatte et al., 2002) but not auditory lists (Hanley & Broadbent, 1987). This is explained within the phonological loop model by supposing that articulatory suppression blocks the access of visually presented memoranda to the phonological store, leaving the irrelevant speech—which gains automatic access to the phonological store—with nothing to interfere with. In contrast, auditorily presented memoranda, like irrelevant speech tokens, gain automatic access to the store and hence are vulnerable to interference from the irrelevant speech despite articulatory suppression (e.g., Baddeley, 2000).

As acknowledged by proponents of the phonological loop model (e.g., Baddeley, 2000; Larsen et al., 2000), however, Salamé and Baddeley’s (1982, 1989) *phonological-interference* account of the irrelevant speech effect faces a number of substantive problems. First, there have been numerous failures to replicate the finding that the irrelevant speech effect is a function of the phonological similarity between the individual speech tokens and the memoranda (Bridges & Jones, 1996; Jones & Macken, 1995b; Larsen et al., 2000; LeCompte & Shaibe, 1997; though see Hughes & Jones, 2005, and Hughes & Marsh, 2017, for evidence that shared phonology at the sequence level exerts an effect). Second, the irrelevant auditory input need not be speech, and hence phonological, at all to disrupt verbal serial recall. For example, Jones and Macken (1993) showed that verbal serial recall is also impaired in the presence of an irrelevant sequence of pure tones changing acoustically from one to the next. Third, not only is the presence of phonology in the sound not necessary for the effect, it is also not sufficient: To observe a pronounced effect, the sound tokens must be changing acoustically from one to the next (e.g., “F, K, R, Q. . .” or a succession of tones varying in fundamental frequency; the *changing-state effect*; Jones et al., 1992; Jones & Macken, 1993). But a steady-state sound sequence, while producing a statistically detectable effect compared with quiet with sufficient power (Bell et al., 2019), produces relatively little disruption, even when it is phonological (e.g., “F, F, F, F. . ..”; Jones et al., 1992). An attempt to accommodate irrelevant nonspeech effects and the changing-state effect within the phonological loop account was made by supposing that any sound—so long as it is acoustically varying—may be sufficiently speech-like (and hence “phonological”) to gain obligatory access to the phonological store. However, if sounds such as an interrupted pitch glide, continuous amplitude-modulated sine tones, a series of sine tones changing in pitch, and broadband noise-bursts varying in band-pass frequency—all of which have been found to disrupt verbal serial recall (Jones et al., 1993; Jones & Macken, 1993; Tremblay et al., 2001)—all gain access to the phonological store, it seems reasonable to question whether it is a store specialised for verbal input at all. Fourth, if the key mechanism of disruption is interference with the representations of the phonemes from which the memoranda are composed, then any verbal STM task should, presumably, be vulnerable to the changing-state effect. However, this is not the case: Only tasks that require or encourage serial order processing—serial recall being the quintessential and most often used example of such tasks—are vulnerable to the changing-state effect (e.g., Beaman & Jones, 1997; Hughes et al., 2007; Jones & Macken, 1993). That is, if changing-state irrelevant sound disrupts the storage or/and retrieval of representations of the individual to-be-remembered items, there should be a changing-state effect regardless of whether those representations are being retrieved for the purpose of reproducing information about individual items or whether they are being retrieved for the purpose of reproducing item-order. Finally, it turns out that, contrary to the findings of Hanley and Broadbent (1987), articulatory suppression eliminates the irrelevant sound effect regardless of whether the memoranda are presented visually or auditorily (Jones et al., 2004). It has been suggested that the irrelevant speech may have impaired serial recall with auditory presentation under articulatory suppression in the Hanley and Broadbent (1987) study only because the speech tokens were presented in the same voice and at the same time as the to-be-remembered items, thereby impeding their encoding at a peripheral, sensory, level (which would be expected to occur regardless of articulatory suppression; Jones et al., 2004; Neath, 2000). Hanley and Bakopoulou (2003) went on to report an additive effect of irrelevant speech and articulatory suppression even when the speech was confined to a retention interval. But a number of studies from the same laboratory (Hanley & Bourgaize, 2018; Hanley & Hayes, 2012; Hanley & Shah, 2012) have since shown that, as conjectured by Jones et al. (2004), this is due to the irrelevant speech producing a suffix effect, whereby irrelevant spoken material following the end of an auditory–verbal list impairs recall of the last few list-items (Crowder, 1971; Nicholls & Jones, 2002). When the probability of the irrelevant speech producing a suffix effect is demoted by presenting the speech in a different voice from the spoken memoranda, articulatory suppression eliminates the irrelevant speech effect (Hanley & Bourgaize, 2018). Thus, as with the phonological similarity effect, engagement in articulatory rehearsal is a precondition for the irrelevant speech/sound effect regardless of presentation modality. As such, the locus of the effect is the articulatory rehearsal process itself, not a passive phonological store.

### The interference-by-process account of the irrelevant sound effect

An alternative account of the irrelevant sound effect posits that it results from interference between the processing of acoustic changes in the sound and the articulatory rehearsal of the memoranda, not by interference within a short-term store. Specifically, when there is change—and only when there is change—between segmentable elements within the sound, information about the order of those elements is automatically encoded as a byproduct of auditory streaming (cf. Bregman, 1990). This involuntary processing of order interferes with the similar but this time deliberate, voluntary, articulatory serial rehearsal process applied to the to-be-remembered items (Hughes & Jones, 2001; Jones & Macken, 1993). This *interference-by-process* account explains not only the changing-state effect itself but also the fact that changing-state compared with steady-state sound only disrupts performance when participants engage in focal serial processing (e.g., Beaman & Jones, 1997, 1998; Hughes et al., 2007; Hughes & Marsh, 2020; Jones & Macken, 1993).

The inextricable relation between auditory perceptual organisation, changing-state sound, and the disruption of focal serial processing is revealed through the fact that the irrelevant sound effect obeys the non-monotonic function discussed earlier between the degree of acoustic change, the resulting likelihood of elements cohering into a single stream, and the accuracy of order perception. For example, if two tones (a, b) presented as irrelevant sound in an alternating fashion (“a, b, a, b, a, b. . .”) are similar enough in pitch to cohere into one single changing-state stream, the expected changing-state effect is observed. When the pitch difference between the two tones is increased somewhat (e.g., “a, **b**, a, **b**, a, **b** . . .”; bold-type used here to denote the increased pitch difference), the changing-state effect is, correspondingly, larger. However, when the two tones are so different in frequency that they are likely to be perceived as two distinct auditory objects—with each object containing one repeating steady-state tone (e.g., “a, **B**, a, **B**, a, **B** . . .”)—the disruption diminishes again (Jones et al., 1999). Such modulation in the degree of disruption supports a pivotal role for auditory streaming in the irrelevant sound effect (see also Hughes & Marsh, 2017; Jones & Macken, 1995a; Macken et al., 2003).

### The primacy-gradient account: A reprieve for the phonological store–based approach to the irrelevant speech/sound effect?

A more recent account of the irrelevant speech/sound effect that sits broadly within a phonological loop framework avoids most of the difficulties faced by the original phonological interference account (Salamé & Baddeley, 1982) by adopting some of the key tenets of the interference-by-process account. Specifically, the primacy-gradient account (Page & Norris, 2003) inherits from the interference-by-process account the notion that the perceptual organisation of changing-state sound automatically yields a representation of order, which in turn impairs a representation of the order of the to-be-remembered items (rather than interfering with phonological representations of the items themselves as in the phonological-interference account). However, a critical remaining difference from the interference-by-process account is that it is still assumed on the primacy-gradient account that the sound interferes with (the representation of order used by) a phonological store, not with articulatory rehearsal. This account is based on the more general primacy model which describes a possible mechanism by which the phonological store represents serial order (Page & Norris, 1998). In this view, serial order (e.g., of a series of items presented for serial recall) is encoded in the form of a primacy gradient of item-activation strengths, where the first item is strongly activated, the second is slightly more weakly activated, and so on across the list. Ordered recall involves an imperfect (or “noisy”) process of trying to select whichever item-representation is most active, outputting it, and then immediately suppressing that representation to avoid its repeated output, and so on in a repeating fashion through the list. It is argued that the presence of irrelevant changing-state sound automatically generates a second primacy gradient which depletes attentional resources required to form the primacy gradient for the to-be-remembered items.

However, note that the ordering mechanism that is disrupted by changing-state sound on this account—the generation of a primacy gradient—is not specific to *verbal* STM nor, therefore, axiomatic to the phonological store concept (see also Caplan et al., 2012). That is, it provides an explanation for why verbal short-term recall is disrupted by irrelevant sound that does not appeal to the defining tenets of the phonological store construct per se but rather appeals to one of several possible ordering mechanisms (see also Burgess & Hitch, 2006; Henson, 1998) that the phonological store might use. Thus, a primacy-gradient mechanism could in principle be attached to an account of the irrelevant sound effect that does not posit the existence of a phonological store (see also “A perceptual-motor approach” section).

Moreover, the assumption within the primacy-gradient account that irrelevant speech impairs serial order processing by depleting attentional resources is at odds with a now relatively large body of work that goes against an attentional-diversion-based approach to the disruptive effects of changing-state speech (e.g., Hughes, 2014; Hughes et al., 2005, 2007, 2013; Hughes & Marsh, 2019, 2020). For example, if changing-state sound depletes attention, then any process that involves attention—not just the formation of a primacy gradient—should be vulnerable to a changing-state effect. But, as noted earlier, only serial order processing is susceptible to a changing-state effect (e.g., Hughes et al., 2007; Hughes & Marsh, 2020; Jones & Macken, 1993). But auditory distraction effects that are universally regarded as being due to attentional diversion—such as that caused by an auditory deviant—are not confined to focal serial order processing (e.g., Hughes et al., 2007).

Finally, the primacy-gradient account, and any other account of the irrelevant speech/sound effect that locates that effect in the phonological store, predicts that there should be an irrelevant speech effect even under articulatory suppression so long as the memoranda are presented auditorily and hence, like the irrelevant speech, gain automatic access to the store. As reviewed above, however, this is not the case (Hanley & Bourgaise, 2018; Jones et al., 2004).

In sum, the irrelevant speech (or more properly “sound”) effect was for a long time considered a cornerstone of the phonological store construct. However, the initial phonological loop-based account of the effect suggested by Salamé and Baddeley (1982) is now generally considered to be untenable (e.g., Baddeley, 2007). More recent attempts to accommodate the irrelevant sound effect within the phonological loop framework (Page & Norris, 2003) do not appeal to the defining characteristics of the phonological store itself. As such, the irrelevant sound effect cannot be taken as positive support for the phonological store construct. In any case, any phonological store–based account of the effect—including the primacy-gradient account—cannot explain the finding that engaging in articulatory rehearsal is a prerequisite for the effect even with auditory presentation of the memoranda. The effect is better understood as reflecting an unwanted confluence between the obligatory perceptual organisation of a changing-state sound sequence and the process of serial articulatory rehearsal (Jones & Tremblay, 2000).

## A perceptual-motor approach

The research discussed in the foregoing sections suggests that the two empirical hallmarks of the phonological store—the phonological similarity effect and the irrelevant speech/sound effect—are better explained by recourse to the action of articulatory planning and the effects of obligatory auditory perceptual organisation, without having to invoke a distinct phonological short-term store. In this section, I elaborate on this alternative, perceptual-motor, approach to verbal serial STM.

### The articulatory plan is a storage mechanism in and of itself

It is worth acknowledging first the substantial debt of gratitude owed by the perceptual-motor account to the phonological loop model for the latter’s role in producing a body of empirical work that has highlighted an important place for articulatory processes in the understanding of verbal serial STM. It is important also, however, to emphasise a fundamental difference in the particular role given to articulatory processes in the two theoretical accounts. Whereas in the original Baddeley and Hitch (1974) account the role of articulatory processing in verbal STM was primary (hence the original term “*articulatory* loop”; cf. Baddeley, 1986), for the reasons discussed in the previous two sections, it has, at least for the past 40 years or so, played a subsidiary role to the passive phonological store (e.g., Baddeley, 1986; Baddeley et al., 1984). As recounted by Baddeley and Larsen (2007), the phonological loop subsystem “was initially termed the articulatory loop, but was renamed the phonological loop, on the grounds that the capacity for storage was the central feature of the system, which can operate without articulation, provided material is presented auditorily” (p. 497). Thus, the current role ascribed to articulatory processes in the phonological loop model is to convert graphemic input so that it can enter the phonological store and, of greater relevance to the present discussion, *articulatory rehearsal* is conceptualised as a process of revivifying representations in the phonological store (which can enter automatically in the case of auditory input) in the face of their rapid decay. Thus, in the latter case, the role of articulatory processes is to counteract a “negative” property (i.e., the decay of individual items) of a separate cognitive structure (i.e., a short-term store). On the perceptual-motor account, in contrast, articulatory processing regains centre stage as the primary means by which a verbal sequence is reproduced (and learned; see below). In this view, such processing, as in the phonological loop model, recodes graphemic input (i.e., with visual–verbal presentation) but into articulatory rather than phonological form. Moreover, articulatory rehearsal is conceptualised not as something that offsets a negative process but as a “forward-acting,” constructive process of articulatory *sequence planning* (e.g., Grush, 2004).

It has often been noted that the lack of a mechanism for the sequencing of the items presented for serial recall was, for a considerable time, a major omission in the phonological loop model (e.g., Baddeley, 2003, 2007; Burgess & Hitch, 2006). Indeed, there is nothing in the architecture of the phonological store that makes it inherently suitable for the retention and reproduction of serial order (for a more extensive discussion of this observation, see Caplan et al., 2012): It holds representations of individual phonemes for around 2 s. And, as discussed, articulatory processing is also not deemed to be involved directly in sequencing (either in the short or long term; e.g., Hitch et al., 2009); it converts visual–verbal items into phonological ones and revivifies the representations of individual phonemes. The reason that the lack of a sequencing mechanism was a major gap in the model is that it had long been argued that the phonological store evolved to retain (and learn) verbal *sequences* (Baddeley, 1986; Baddeley et al., 1998). Indeed, the phonological store construct has been built empirically on the basis of serial recall tasks in which the sole (or at least primary) burden is to reproduce the *order* of a list of items, that is, where the individual items themselves are already known to the participant before the list is presented (e.g., a sequence of digits in a random order) or soon become known through exposure to a restricted set (e.g., of letters or words) used repeatedly (in different orders) across an experiment. Thus, the putative empirical hallmark of the phonological store—the phonological similarity effect—for example, specifically impairs item-order memory and indeed phonological similarity typically has the opposite effect on item memory; it enhances it (Nimmo & Roodenrys, 2006; Wickelgren, 1965). The solution to this omission has been to attach separate models of serial order onto the phonological store concept (see, e.g., the primacy model discussed briefly in the previous section, Page & Norris, 1998; see also Burgess & Hitch, 2006; Henson, 1998). But none of these are axiomatically phonological store–based models: The phonological store may make use of these mechanisms but they are not distinctly phonological sequencing mechanisms (Caplan et al., 2012; Page et al., 2006).

A process, or skill, that is indeed inherently sequential, however, is speaking and (hence) speech planning. Thus, rather than articulatory processing being seen as a means to offset a “negative” characteristic of a separate (phonological) store—the decay of the representations of individual phonemes—on the perceptual-motor account, the act of articulation (or more accurately, subvocal articulatory planning) is the very means by which the typical serial recall list is turned into, retained, and reproduced as a sequence. In short, notwithstanding the additional influence that obligatory auditory perceptual-organisation processes can have on auditory–verbal serial recall, the articulatory plan *is* the storage mechanism, not something that supports a separate storage mechanism.

An important starting point here is the characterization of the typical serial recall list as a list of items that are semantically unrelated to one another and grammatically and syntactically unconstrained: The serial recall researcher would not typically present a list such as “Mary, had, a, little, lamb” but rather an unrelated list of words, digits, or letters that do not, ideally, have pre-experimental sequential associations (e.g., 3, 1, 2. . . or B, C, A. . . might be presented but not 1, 2, 3. . . or A, B, C. . .). That is, the transitional probability (cf. Miller & Chomsky, 1963) between successive items in the typical serial-recall list is, by design, low. In the face of the low transitional probabilities to be found in the standard serial recall list, the skill of (sub)articulatory sequencing is exploited opportunistically to increase those probabilities, that is, to bind the items into a temporally extended (motor) object that will serve as the basis of reproduction (whether the response is ultimately to vocally output the list, to write it down, or reconstruct it via mouse-clicking on the items in the correct order and so on).

One way in which articulatory planning generates information that is not present in the list itself and which serves to bind the items over time is through the sub-skill of coarticulation (Hardcastle & Hewlett, 2006; Sternberg et al., 1980). This refers to the fact that the precise manner in which one speech element (e.g., phoneme, syllable, word) is (subvocally) spoken differs as a function of the identity of its neighbour: The coarticulation of the two elements thereby creates a new compound object that embodies information about the order in which the successive elements occurred. For example, if “one” is followed by “three” (e.g., in a digit span task), the mere act of articulating them one after the other provides information that binds them: Whereas the offset of “one” (the sound /*n*/) typically has an alveolar place of articulation (when followed, say, by the utterance “four”), when it is followed by “three,” its place of articulation adjusts to become dental so as to ease the transition to the dental /θ/ at the onset of “three.” The effect that such coarticulatory habits has on verbal serial recall performance has been demonstrated by showing, for instance, that practice at coarticulating a particular set of verbal items improves the recall of those items over and above any enhancement attributable to increased familiarity with the individual items themselves (Woodward et al., 2008). In addition, digit span in Welsh is not lower than in English because the individual digit-words are longer in Welsh (cf. Ellis & Hennelly, 1980)—indeed, they are shorter on average—but because the transitions between them are more complex (A. Murray & Jones, 2002). This effect can also be demonstrated by constructing two sets of words within a language (English): One where the transitions are complex due to a requirement to change the place of articulation at each word boundary (e.g., *tape, knife, turf*. . .) and one where they are less complex due to little or no need to change the place of articulation (e.g., *nurse, wren, sill. . .*); serial recall is significantly poorer for the difficult-to-coarticulate words (A. Murray & Jones, 2002).

The natural prosodic features of speech (and hence speech planning) also imbues a list with information that constrains the order of its elements. When a serial recall list is presented in a temporally grouped fashion (e.g., *F, H, K,. . .Q, R, Y*), the overall accuracy serial position curve is made up of a number of micro, “scalloping,” serial position curves, one relating to each group. Overall accuracy is also enhanced appreciably because the belongingness of items to a particular group reduces order errors (transpositions) between immediately successive items located at the group boundaries (e.g., *K* and *Q* in the above example), although there is also a (lesser) cost due to an increased likelihood of remote transpositions between items occupying the same position within different groups (e.g., *F* and *Q* in the above example) (which, as discussed earlier, is also seen in naturally occurring articulatory-planning errors). Moreover, the timing of the output of such lists also qualitatively mimics the presented grouping (Maybery et al., 2002; Sjöblom & Hughes, 2020). That this grouping-based enhancement of recall is driven at least in part by articulatory planning (at least with visual presentation where there is no automatic perceptual-organisation-based grouping; see Frankish, 1985, 1989; Sjöblom & Hughes, 2020; see also Ryan, 1969) is supported by the finding that both the grouping-based modulation of the serial position curve and the grouping-based output-timing pattern is eliminated or at least markedly attenuated under articulatory suppression (Hitch et al., 1996; Sjöblom & Hughes, 2020). Importantly, there is evidence that such articulatory grouping and its concomitant effects on serial recall also occurs spontaneously, that is, even when the list itself (i.e., as presented) is not grouped (Wickelgren, 1964, 1967). This strongly suggests a natural tendency during serial recall to exploit the prosodic nature of speech and speech planning to constrain the order of items in preparation for output.

In addition to its main emphasis on articulatory planning, the perceptual-motor account also, as noted throughout much of the discussion thus far, reconceptualises certain key phenomena of serial recall found with auditory material (both task-relevant and task-irrelevant) as reflecting the action of automatic, acoustic-based, auditory perceptual organisation that proceeds independently of the articulatory system. The development of this aspect of the perceptual-motor account also owes a great deal to the phonological loop model insofar as the latter model was one of the first to fully highlight the fact that presentation modality (auditory vs. visual) needs to be taken into account in the understanding of verbal serial STM (e.g., Baddeley et al., 1984; Vallar & Baddeley, 1984). However, once again, there is a fundamental difference in the particular way in which presentation modality is taken into account in the two approaches: As discussed earlier, in relation to the phonological loop model—or more historically accurate, the articulatory loop model—a phonological store was added primarily to accommodate the fact that certain apparently phonological effects on serial recall performance (e.g., the phonological similarity effect) were still found with auditory, but not visual, presentation despite the incapacitation of the articulatory system by articulatory suppression (Baddeley et al., 1984). On the perceptual-motor account, in contrast, serial-recall effects that are unique to auditory (compared with visual) presentation are explained by reference to the way in which the auditory perceptual system, unlike the visual-perceptual system (which is concerned primarily with organisation of its inputs in space), inherently organises its inputs *sequentially* in the form of temporally extended perceptual objects or *streams* (for an extensive discussion, see Bregman, 1990).

## A (damaged) phonological store in the brain?

I have argued thus far that the key serial recall effects observed in neurologically unimpaired participants that have been taken as support for a passive phonological store do not in fact provide such support and are better explained in terms of the action of articulatory and perceptual processes. Often cited as strong additional support for the phonological store concept, however, are neuropsychological case studies of brain-damaged individuals who are argued to have a selective deficit of the passive phonological store. Of key interest here are rare “short-term memory patients,” individuals with aphasia arising from damage to the inferior region of the left parietal lobe, and more specifically the supramarginal gyrus (SMG). These patients—around 20 of which had been identified as of 2019 (Shallice & Papagno, 2019)—were said to exhibit a selective difficulty with auditory–verbal serial recall in the absence of any clear perceptual, language, or articulatory difficulties, leading to the conclusion that they have a defective phonological store (e.g., Shallice & Papagno, 2019; Vallar & Baddeley, 1984; Vallar & Papagno, 2002; Warrington & Shallice, 1969, 1972).

However, the pattern of performance exhibited by STM patients is, as Vallar (2006) summarises, “comparable to that shown by neurologically unimpaired individuals when engaged in articulatory suppression” (p. 140; see also Vallar & Papagno, 2002). In other words, much of the pattern of performance they show in verbal STM tasks is consistent with a problem with articulatory planning/rehearsal rather than passive phonological storage. First, span (or serial-recall performance) is, of course, low, in these patients, just as it is markedly lower when articulatory processing is restricted in neurologically unimpaired individuals by articulatory suppression (Baddeley, 1986; D. J. Murray, 1968). Second, the majority of such patients show a phonological similarity effect with auditory but not visual presentation (e.g., Vallar et al., 1992), just as neurologically unimpaired participants do under articulatory suppression (Baddeley et al., 1984; though see “The phonological similarity effect” section above for evidence that this is an acoustic, not phonological, similarity effect; Jones et al., 2004). Third, the poorer recall of a list of long compared with short words (the word-length effect)—the classic hallmark of the involvement of articulatory processes in verbal STM tasks according to the phonological loop model (Baddeley et al., 1975)—is absent regardless of presentation modality, again just as it is in neurologically unimpaired participants under articulatory suppression (Vallar & Papagno, 2002). In addition, articulatory suppression does not further impair visual–verbal recall performance in these patients (e.g., Vallar & Baddeley, 1984), as would be expected if their neurological damage is already effectively “suppressing” the use of articulatory processes. A key defining feature of the STM patient’s performance, however, that differs from that of neurologically unimpaired participants under articulatory suppression is that their recall of visually presented verbal lists is relatively well preserved compared with that of auditorily presented verbal lists and, moreover, in at least some such patients, their visual–verbal recall performance shows evidence of the use of visual-based rather than phonological or articulatory strategies (e.g., Warrington & Shallice, 1972). In addition, the deficit in auditory–verbal serial recall in the STM patient is particularly marked, and sometimes only present, at recency (the last one or two items in a list; e.g., Basso et al., 1982; Saffran & Marin, 1975; Vallar et al., 1997).

Based on this pattern of data, Vallar (2006) suggests that “the process of *[articulatory] rehearsal* is *either primarily damaged*, or unimpaired but not utilised by these patients. . .[t]here may be no advantage in rehearsing items held in a damaged store” (emphasis added, p. 140; see also Vallar et al., 1997; Vallar & Papagno, 2002). It is important to emphasise just how different the implications of these two distinct hypotheses are: Accepting Hypothesis 1—“the process of [articulatory] rehearsal is primarily damaged”—would mean that the patient data would not provide strong evidence for the passive phonological store concept because, as witnessed in the form of the perceptual-motor account, for example, assuming a key role for articulatory processing in verbal STM does not in itself compel the postulation of a phonological store. Rather, the phonological store concept only enjoys support from the neuropsychological data if Hypothesis 2 is correct: That the problem is not with the articulatory planning/rehearsal system itself but rather due to selective damage to (or the absence of) a passive phonological store, which then, in turn, discourages the use of (an intact) articulatory rehearsal system.

An immediate difficulty for Hypothesis 2, however, is that the majority of STM patients, as noted, show a phonological similarity effect with auditory presentation (Vallar et al., 1992; Vallar & Papagno, 2002). Thus, it seems that either the phonological similarity effect is not, after all, the signature of the use of a phonological store or that these patients have an intact (or at least relatively spared) phonological store (Caplan et al., 2012). Indeed, as discussed in a previous section, it is precisely the presence of a phonological similarity effect with auditory but not visual lists (under suppression in neurologically unimpaired participants) that formed the main basis of the postulation of a passive phonological store separate from articulatory processes in the first place (Baddeley et al., 1984).

The rejection of Hypothesis 1 by proponents of the phonological loop model and their championing of Hypothesis 2 (e.g., Baddeley, 2003; Vallar, 2006)—despite the evidence just noted suggesting at least a relatively spared phonological store—appears to be based on the following pieces of evidence and lines of reasoning: First, many of the patients show evidence of normal spontaneous speech production (as well as normal speech comprehension; Patient T.B., Baddeley et al., 1987; Baddeley & Wilson, 1988; Patient I.L., Saffran & Marin, 1975; Patient J.B., Shallice & Butterworth, 1977; Patient P.V., Vallar & Baddeley, 1984). The logic here, then, appears to be that given that spontaneous speech is normal, the deficit in auditory–verbal recall is unlikely to be attributable to a difficulty with articulatory planning/rehearsal. However, this logic is, in my view, unsound: The degree of articulatory planning required to (re)produce a *novel* sequence such as in a serial recall task is very different from the degree of planning required to produce a normal phrase or sentence, which is scaffolded by long-term syntactic and grammatical knowledge that makes the transitional probabilities between successive elements of the plan relatively high. As discussed earlier, on the perceptual-motor account, it is precisely when transitional probabilities are low—such as is deliberately the case in a serial-recall list—that great demands are made on the articulatory-planning system to bind the otherwise successively unrelated elements together into a (motoric) sequence. Thus, the presence in the STM patient of normal spontaneous speech production or normal performance in “simple” tests of speech production (e.g., picture naming) or of phonological judgement does not rule out a problem with articulatory planning when faced with a STM task. And indeed, there is evidence that the STM patient does have difficulties with producing (and comprehending) long or/and complex, sentences (e.g., Vallar & Baddeley, 1984; Warrington & Shallice, 1969), that is, with sequences that begin to take on the characteristics of the typical serial recall list. Of course, the likely and perfectly legitimate counterpoint from the perspective of the phonological store account would be that reproducing a serial recall list or comprehending or producing a complex sentence is precisely when the services of a phonological store are needed (e.g., Vallar & Papagno, 2002). Nonetheless, it can be suggested that evidence pointing to intact natural speech and language processing and spared performance in relatively simple speech and language tests is not sufficient to rule out Hypothesis 1 (i.e., an articulatory-planning deficit).

Another argument that has been forwarded against an articulatory *production* deficit at least is that, in many STM patients, performance does not improve when a nonspeech response is required, such as when participants must instead point to the correct items in sequence in a multiple-choice display (Patient P.V., Basso et al., 1982; Patient K.F., Warrington & Shallice, 1969), or when memory is tested via a matching task (Patient E.D.E., Berndt et al., 1991; and Patients K.F., J.B., and W.H., Warrington et al., 1971) or a probe recognition method (Patient K.F., Shallice & Warrington, 1970). There are a number of other cases, however, who do show a marked improvement in performance in auditory–verbal recall when a speech response is not required (Patient R.L., Caplan et al., 1986; Patient J.O., Kinsbourne, 1972; Patient G.C., Romani, 1992; Patient T.O., Vallar et al., 1997), suggesting that the deficit in these particular cases is indeed located, at least in part, in the speech production process (Vallar, 2006; Vallar & Papagno, 2002). Moreover, even though such methods do not require a vocal response, this does not speak to whether or not subvocal articulatory planning/rehearsal is still usually used (by a neurologically unimpaired participant) to support performance. Indeed, the vast majority of verbal serial recall studies, at least with neurologically unimpaired participants, do not involve a vocal response either: Responding is typically written or via order reconstruction (effectively “pointing”). Thus, the impaired performance of those patients who do not show a benefit when the response mode is non-articulatory may still, in principle, be suffering from an articulatory planning/rehearsal deficit. For example, auditory–verbal matching (or serial recognition) tasks—in which two successive verbal sequences are to be judged as being the same or slightly different from one another (usually via keypress)—especially with the relatively slow presentation rates (e.g., 1 item per second; Warrington et al., 1971) typically used with brain-damaged patients, is likely to be supported by articulatory planning/rehearsal (see B. Macken et al., 2014; Warren, 1999). Articulatory planning/rehearsal may also be involved in probe recognition tasks in which, for example, the participant must state (e.g., via keypress) whether or not a single probe item (“K”) appeared in a just-presented list (“F, R, K, T, Q”; Shallice & Warrington, 1970). More generally, there are many examples of findings that caution against assuming that a task does not invoke an articulatory serial rehearsal strategy simply because it does not call explicitly for the processing or reproduction of serial order (e.g., Beaman & Jones, 1997, 1998; Bhatarah et al., 2009; Hughes & Marsh, 2020; B. Macken et al., 2014).

In sum, the qualitative pattern of performance found in STM patients largely mimics that of neurologically unimpaired participants under suppression, suggesting an articulatory planning/rehearsal deficit (Hypothesis 1). The fact that these patients can often exhibit normal spontaneous speech production or normal performance in simple tests of speech production or phonological judgement, and the fact that not all of them show a benefit from using a non-articulatory response mode, does not seem to warrant the rejection of this hypothesis in favour of the alternative, phonological storage deficit, view (Hypothesis 2). I return, therefore, to Hypothesis 1 according to which the deficit is related to an articulatory planning/rehearsal problem, in line with the perceptual-motor approach.

The main challenge for Hypothesis 1, perhaps, is to explain the selectivity of the deficit to *auditory–*verbal (as opposed to visual–verbal) stimuli. However, here I tentatively suggest an addition to Hypothesis 1—which I will call Hypothesis 1.1—based on the perceptual-motor account that might also explain this selectivity: Due to the fact that passive auditory perceptual-organisation processes lead to the automatic serial encoding of such items (e.g., Bregman, 1990; Jones & Macken, 1995a; Warren, 1999), the STM patient is, with auditory lists, seduced to use an articulatory strategy to convert those automatically ordered items into articulatory form for the purpose of potential overt reproduction. However, given that the articulatory-planning system is damaged, this strategy largely fails, resulting in poor performance. Due to the fact that with visual–verbal lists, in contrast, there is no automatic encoding of order, there is little or no temptation to attempt an articulatory strategy and the patient is thus more free to turn to other, non-articulatory, strategies that rely on processes that are relatively intact (visual-based strategies, e.g., Warrington & Shallice, 1972).

Hypothesis 1.1 appears to capture many of the more detailed aspects of the pattern of neuropsychological data too: It explains the phonological similarity effect with auditory but not visual presentation because this would be an acoustic similarity effect that would not be expected to be affected by damage to articulatory processes (cf. Jones et al., 2004, 2006). A word-length effect would not be expected regardless of presentation modality because, on the perceptual-motor account (as well as the phonological loop model), this effect is a product of articulatory processing. Finally, as noted, there is some evidence that the loss of auditory–verbal serial-recall performance in the STM patient is particularly pronounced at recency (Basso et al., 1982; Vallar et al., 1997), precisely where automatic acoustic-based order encoding is particularly strong (e.g., Jones et al., 2004; Maidment & Macken, 2012; Nicholls & Jones, 2002) and hence where the temptation to try to map that encoding onto a (defective) articulatory system may also, therefore, be particularly strong. At the same time, the greater prominence of the deficit at auditory recency appears to present a further difficulty for the phonological store-deficit hypothesis (Hypothesis 2) given that, as discussed earlier, auditory recency (or the modality effect) is said to lie outside the explanatory compass of the phonological store concept (Baddeley, 1986; Baddeley & Larsen, 2007; Hurlstone et al., 2014; Page & Norris, 1998).

Could the phonological loop model, however, also effectively adopt Hypothesis 1.1? In this view, the articulatory rehearsal process is damaged but it is the intact passive phonological storage (rather than intact automatic acoustic-based perceptual organisation) that tempts patients to use that damaged articulatory system with auditory (but not visual) lists. There are two main difficulties with this idea. First, if it is articulatory processing that is defective, then while the neuropsychological data may still be consistent with the phonological loop model, they no longer provide specific direct support for the existence of a passive phonological store. That is, the reasoning has been, classically, that the fact that the passive phonological store can be selectively impaired or destroyed due to brain damage provides strong evidence for such a store. However, if instead it is articulatory rehearsal that is damaged, one need not necessarily invoke a passive phonological store at all. A second difficulty is that the phonological loop model would seem to be compelled to reject Hypothesis 1.1 for the same reason that it rejects Hypothesis 1, namely, that spontaneous speech production and performance in simple tests of speech production and of phonological judgement appear to be normal (Vallar, 2006). The reason that these findings suggest that the articulatory component of the phonological loop model is intact can be traced back to the function that the model ascribes to articulatory processes, that is, to recode individual visual items into phonological form and to reactivate the phonological representations of individual memoranda (regardless of presentation modality). Thus, if spontaneous speech production is possible, there is little reason to suppose that such item-recoding and item-reactivation would be impaired. As such, the evidence pertaining to intact speech production makes it difficult for the phonological loop model to adopt Hypothesis 1.1: It would not be clear why, on the phonological loop model, the STM patient fails to show the hallmarks of rehearsal: A word-length effect (regardless of presentation modality) and a phonological similarity effect with visual (and not just auditory) lists. A third difficulty has already been noted, namely, that the deficit in auditory–verbal serial recall is particularly pronounced at recency, to which the phonological store does not contribute (Baddeley, 1986). However, this is a difficulty for any phonological store-based account of the neuropsychological data, not only for its possible adoption of Hypothesis 1.1.

Another argument that has been made against Hypothesis 1 (and which would apply also to Hypothesis 1.1.) and in favour of Hypothesis 2 is that other brain-damaged patients who are claimed by proponents of Hypothesis 2 to indeed have a defective articulatory system (but an intact phonological store) have different behavioural and neuroanatomical profiles from those of the STM patient (e.g., Patient T.O., Vallar et al., 1997). The evidence that most of these patients suffer from an articulatory rehearsal deficit is convincing (Vallar & Papagno, 2002). Indeed, one interpretation of the behavioural profile of such patients that would be in line with Hypothesis 1.1 is that their articulatory deficit is simply more extreme than that of the STM patient. In this view, while the STM patient only shows evidence of articulatory-planning difficulties when the demand on articulatory planning is particularly high—such as when needing to recall a serial recall list or a complex sentence but not when producing spontaneous (relatively simple) sentences—the rehearsal-deficit patient also has difficulties with “everyday” (relatively undemanding) speech production, as reported, for example, by Vallar et al. (1997). Thus, if the impairment of articulatory processes found in the rehearsal-deficit patient can indeed be interpreted as a more extreme version of the articulatory-planning deficit that Hypothesis 1.1. assumes in the case of the STM patient, then the behavioural case for a dissociation between the STM patient and the rehearsal-deficit patient rests on the latter having an intact phonological store while the former has a damaged phonological store. But the evidence for this seems weak.

The most direct test of the predicted critical double dissociation between the STM patient and the rehearsal-deficit patient comes from a study by Vallar et al. (1997), who contrasted case T.O. (classed as a rehearsal-deficit patient) and case L.A. (classed as an STM patient). Suggesting that T.O. had an intact phonological store, Vallar et al. (1997) found that he showed a phonological similarity with auditory (but not visual) lists. The difficulty here is that, as noted earlier, the vast majority of STM patients also show a phonological similarity effect with auditory (but not visual) lists (Vallar & Papagno, 2002). The fact that Vallar et al.’s (1997) particular STM patient, L.A., happened not to show a phonological similarity effect with auditory (or visual) lists does not, therefore, dissociate the rehearsal-deficit patient from the STM patient generally; rather, it suggests that L.A. is a rather atypical STM patient. Indeed, not only is L.A.’s profile inconsistent in some ways with the STM patient, it is also difficult to interpret generally: For example, L.A. (like the classic STM patient) did not show a word-length effect—suggesting no use of articulatory rehearsal—but did (unlike the classic STM patient) show an effect of articulatory suppression, suggesting that they did indeed engage in articulatory rehearsal (in no-suppression conditions). Patient T.O., as expected, did not show either of these effects.

A second finding cited by Vallar et al. (1997) as suggesting that T.O. has an intact phonological store is that he showed, in the context of an auditory–verbal serial recall task, an irrelevant speech effect. This was interpreted as being consistent with a spared phonological store because, on the phonological loop model, irrelevant speech specifically impairs phonological storage. On the perceptual-motor account, in contrast, little or no irrelevant speech effect would be expected in this case because, as discussed earlier, this account locates this effect in the articulatory rehearsal process (Jones et al., 2004), which is defective in T.O. On the face of it, then, the finding that T.O. was vulnerable to irrelevant speech supports the phonological store-based account over the perceptual-motor account. However, the “irrelevant speech effect” in this case may have been a spurious one: Vallar et al. (1997) compared the effect of continuous, relatively loud [75 db(A)], changing-state irrelevant speech played throughout the presentation and recall of auditorily presented to-be-remembered lists compared with a quiet control condition. It is therefore difficult to be sure that this was a “true” irrelevant speech effect: It may have been a sensory masking effect (e.g., Hanley & Broadbent, 1987), a difficulty of perceptual partitioning (Nicholls & Jones, 2002), a suffix effect (Hanley & Bourgaize, 2018), a general attentional distraction effect (e.g., Hughes, 2014; Hughes & Marsh, 2020), or some combination of these. To ascertain whether T.O. (and other rehearsal-deficit patients) exhibits a classical irrelevant speech/sound effect and rule out these alternative explanations, one would need to (1) ensure that the speech does not affect the perceptual encoding of the spoken memoranda (e.g., by capitalising on principles of auditory streaming; see Jones et al., 2004); (2) have the speech/sound cease at the same time as the to-be-remembered list; (3) include a steady-state speech control condition; and, ideally, (4) add a control task that is unlikely to involve or encourage a serial rehearsal strategy (e.g., Hughes et al., 2007).

A third observation that has been claimed to demonstrate that T.O. (and other rehearsal-deficit patients) has an intact phonological store is that they show normal recency during a free recall task (Vallar et al., 1997). However, it is difficult to make the case generally that the phonological store contributes to recency in free recall because recency in this task does not show a classic phonological similarity effect (while this has not, to my knowledge, been tested in the context of neuropsychological cases, for relevant studies of neurologically unimpaired participants, see, for example, Baddeley, 1976; Craik & Levy, 1970; Glanzer et al., 1972; Watkins et al., 1974; see also Richardson & Baddeley, 1975). Indeed, in the seminal Baddeley and Hitch (1974) paper, it was suggested that working memory “has access to phonemically coded information (possibly by controlling a rehearsal buffer), that it is responsible for the limited memory span, *but does not underlie the recency effect in free recall*” (p. 86, emphasis added).

A further difficulty for the view that the rehearsal-deficit patient has, unlike the STM patient, an intact phonological store is that their performance does not dissociate in relation to the core, defining, characteristic of the STM patient: Both types of patients exhibit greater difficulty with *auditory–*verbal recall compared with *visual–*verbal recall (e.g., Vallar et al., 1997). To recap, the basis of the phonological store-deficit account of the STM patient’s profile—that they strategically opt not to use (an intact) articulatory rehearsal process because of a defective phonological store (i.e., Hypothesis 2)—rests on the relatively intact *visual*–verbal recall performance in these patients. Specifically, recall of *visual–*verbal lists is said to be relatively spared in these patients because such lists uniquely afford the use of visual-based strategies and the recall of such lists does not therefore rely as much on the (damaged) phonological store as does auditory–verbal recall. The fact that the rehearsal-deficit patient—who is presumed to have an intact phonological store—shows the same selective difficulty with *auditory–*verbal recall undermines the logical basis of Hypothesis 2. Therefore, it is unclear why a rehearsal deficit would affect auditory–verbal recall more than visual–verbal recall. Indeed, given the obligatory access of auditory–verbal input into the (intact) phonological store, the opposite should, if anything, be the case. The selective difficulty with auditory–verbal recall common to both types of patients is compatible with Hypothesis 1.1 however: In both cases, there is a difficulty with articulatory planning which only manifests with auditory–verbal input because only such input automatically generates a representation of order that tempts the patient to use their (defective) articulatory-planning system.

Another potential argument against Hypothesis 1.1 and in favour of Hypothesis 2 could be based on the fact that the anatomical location of the main site of damage in most “short-term memory patients”, namely, the left inferior parietal region, and more specifically the SMG, contrasts with the fact that the damage in most rehearsal-deficit patients is in Broca’s area (BA 44), premotor cortical regions (BA 6), and the supplementary motor area (Vallar, 2006).

However, the SMG, the supposed site of the passive phonological store, has also been implicated in active articulatory planning: Brain imaging methods have shown that the area has reciprocal connections to the ventral premotor cortex and inferior frontal gyrus (IFG; pars opercularis) regions, which are typically associated with articulatory planning (Catani et al., 2005; Petrides & Pandya, 2009; Rushworth et al., 2006). Moreover, while the SMG is implicated in “phonologically” demanding tasks, functional magnetic resonance imaging (fMRI) activation of the SMG during rhyme (Petersen et al., 1988), syllable (Devlin et al., 2003; Price et al., 1997), and phoneme judgements (Raizada & Poldrack, 2007; Zevin & McCandliss, 2005) has been argued to be due more to the articulatory requirements of those tasks than to any requirement to store abstract verbal representations (Pattamadilok et al., 2010). Other imaging studies have implicated the SMG in the process of reading (Jobard et al., 2003) and still others suggest that the area is involved in motor behaviours beyond vocal-articulatory ones too, such as visually guided hand actions (Binkofski et al., 2004; Price, 2010; Rushworth et al., 2001). In short, damage to the SMG could affect verbal STM by impairing articulatory planning, not passive phonological storage. Indeed, brain imaging research has not been able to identify any region in the parietal lobe (or indeed any lobe) that exhibits the properties that would be needed for it to be identified with the cognitive concept of a phonological store (for extensive discussions, see Buchsbaum & D’Esposito, 2008, 2019). It seems possible that, from the standpoint of Hypothesis 1.1., the (more extreme) articulatory difficulties in rehearsal-deficit patients results from damage to different “articulatory” areas (e.g., supplementary motor area), ones that are particularly important for overt production and not just subvocal planning (MacNeilage, 1998).

In sum, the neuropsychological (and neuroscientific) data do not provide any clear support for the existence of a passive phonological store and indeed the evidence seems more consistent—or at least just as consistent with—the hypothesis that the “short-term memory patient” suffers from a deficit of articulatory planning rather than a deficit of passive phonological storage.

## But isn’t a phonological store needed to learn new words?

Soon after the introduction of the concept of the phonological store, it became unclear what the evolved function of the store might be when it was discovered that many of the STM patients that were suggested to have a selective deficit of the phonological store (see previous section) suffered little in terms of everyday cognitive functioning (Vallar & Baddeley, 1987). However, a possible resolution to this quandary came when it was discovered that some STM patients were impaired in their ability for long-term verbal sequence learning (Baddeley et al., 1988). Thus, the current view is that the phonological store evolved as a language-learning device (hereafter termed the Phonological Store as Language-Learning Device, or PS-LLD, hypothesis); more specifically, it supports the learning of the phonological-forms of new words, a fundamental building-block of language acquisition both for the infant learning their native language and for the second-language learner (Baddeley & Hitch, 2019; Baddeley et al., 1998).

The key finding that first led to the development of the PS-LLD hypothesis is that some STM patients were found to be able to learn new pairs of real words but not learn word–nonword (or known-word—foreign-word) pairs, that is, they were impaired in their ability to learn new phonological sequences (Baddeley et al., 1988; Papagno & Vallar, 1995). It has been argued that the fact that new word-form learning in the context of this (word-nonword) paired-associate learning task is impaired when the nonwords are relatively long, under articulatory suppression, and when the nonwords are phonologically similar to one another (Papagno & Vallar, 1992) supports the involvement of a phonological store in word-form learning (Baddeley et al., 1998). However, this inference can be questioned on the grounds that on the phonological loop model (as well as the perceptual-motor account), detrimental effects of word length and of articulatory suppression are taken as evidence for the action of articulatory processes, not of (or only indirectly of) passive phonological storage (Baddeley, 1986, 2007). Moreover, the evidence reviewed earlier indicates that the phonological similarity effect is also primarily a product of articulatory processing (e.g., Jones et al., 2004, 2006). Thus, whilst there is clear evidence of a role of articulatory planning in word-form learning in this task, it is unclear what the evidence is for a role of passive phonological storage over and above such articulatory processes. It has sometimes been suggested that the fact that (some) STM patients have difficulty with word–nonword paired-associate learning is itself evidence of the involvement of the phonological store in such learning (Baddeley, 2021). However, the veracity of this inference is of course predicated on the assumption that the STM patient has been correctly identified as suffering from a selective deficit of a phonological store in the first place, an assumption that is, as argued in the previous section, open to challenge.

There also appears to be a contradiction between inferences drawn from the paired-associate learning paradigm regarding the role of the phonological store in word-form learning and those drawn more recently from the Hebb repetition paradigm (Hitch et al., 2009; Page et al., 2006). The Hebb repetition effect, or Hebb sequence learning, refers to the enhanced recall of a serial recall list that is intermittently re-presented every few trials (Hebb, 1961). Several proponents of the phonological loop model have capitalised on this effect as a convergent means of investigating the PS-LLD hypothesis (Burgess & Hitch, 2005; Hitch et al., 2009; Norris et al., 2018; Page et al., 2006). Some of the key findings from this endeavour include the observation that Hebb sequence learning is immune to articulatory suppression (Hitch et al., 2009; Page et al., 2006) and to phonological similarity (Hitch et al., 2009). It has been argued that the absence of these effects on Hebb verbal sequence learning supports the PS-LLD hypothesis on the grounds that articulatory suppression and phonological similarity only affect item-level (or sub-item-level) representations in the phonological store (which will impair *short-term* recall) and not the (separate) representation of order within the store that supports long-term sequence learning. However, this means that, on one hand, the absence of an effect of articulatory suppression and phonological similarity on phonological sequence learning (in the Hebb repetition task) supports the PS-LLD hypothesis but, on the other hand, the presence of an effect of these same variables on phonological sequence learning (in the paired-associate task) also supports the PS-LLD hypothesis. It is difficult to see how both inferences could be valid.

However, more recent evidence suggests that the conclusion that Hebb sequence learning is unaffected by articulatory suppression was, in any case, premature: In Sjöblom and Hughes (2020), we found that articulatory suppression does indeed abolish or at least dramatically impair such learning. We also found that phonological similarity modulates Hebb sequence learning: It enhances it (contrary to previous assumptions that it might, if anything, impair it; Hitch et al., 2009) because, we suggested, the recall of a relatively difficult-to-recall sequence (a phonologically similar list) has more to gain from repeated practice (cf. Newell & Rosenbloom, 1981). We argued, based on the perceptual-motor account, therefore, that Hebb sequence learning is driven largely by the repeated active articulatory planning of the repeating sequence, not its repeated passive phonological storage. Further evidence for an articulatory account came from the finding that an inconsistent temporal grouping of the list-items across instances of the repeating list (e.g., *F, H, K,—L, Q, R, Y* on first presentation and *F, H, K, L—Q, R, Y* on the second) also attenuated learning, but only when the inconsistent grouping effect was driven by temporally inconsistent articulatory plans; the inconsistent grouping effect was abolished under articulatory suppression.

Thus, the evidence from paired-associate learning and Hebb verbal sequence learning in fact converges but not on the conclusion that learning in each case reflects the action of a passive phonological store but that learning in both settings reflects the legacy of the short-term articulatory planning of the sequence. Learning in the paired-associate learning task is modulated by articulatory suppression, word length, and phonological similarity (which has, on the perceptual-motor account, been reascribed to articulatory-planning errors; see “Phonological similarity effect” section) and Hebb sequence learning is modulated by articulatory suppression, phonological similarity, and temporally inconsistent articulatory planning.

### Nonword repetition

Another line of evidence cited as strong support for the PS-LLD hypothesis is the positive correlation between nonword repetition (NR)—the ability to immediately repeat an auditorily presented nonword (e.g., “woogalamic”)—and vocabulary size, both in children and in adults (e.g., Gathercole, 2006; Gathercole & Baddeley, 1989; Gathercole et al., 1999). Key to the argument that this provides strong evidence for the PS-LLD hypothesis is the claim that NR performance constitutes a particularly pure measure of the passive phonological store, one uncontaminated by the involvement of articulatory rehearsal: “Nonword repetition provides a measure of the phonological store, not phonological rehearsal” (Baddeley et al., 1998, p. 168). Thus, the correlation between NR and vocabulary size is, accordingly, seen as directly measuring the capacity and evolved function of the phonological store (Baddeley et al., 1998). Specifically, the PS-LLD hypothesis holds that the function of the passive phonological store is to temporarily retain a novel sequence of phonemes (i.e., a “new word”) while a long-term representation of it is formed.

The notion that NR is supported by a phonological store appears to be inferred from the assumption that a phonological store supports verbal serial recall together with similarities between verbal serial recall and NR (e.g., performance on the two tasks is correlated; they produce comparable serial position curves; and show similar grouping and item-length effects; Gupta, 2005; Gupta et al., 2005). However, none of these lines of evidence necessarily indicate that NR is supported by a passive phonological store because they could plausibly reflect the common involvement of articulatory processes in the two tasks. Indeed, contrary to the critical notion that NR performance provides a relatively pure index of passive phonological storage, we have shown recently that NR is markedly impaired by articulatory suppression (but not by concurrent tapping; Hughes et al., 2024). Similarly, NR shows a nonword-length effect (Archibald et al., 2009). Given that on the phonological loop model, an item-length effect in serial recall reflects the role of articulatory rehearsal in performance, it is unclear why the same effect does not indicate a role for articulatory processes in NR.

The rejection of the notion that articulatory processing plays a role in NR—or in the correlation between NR and vocabulary acquisition—appears to be based primarily on the fact that the critical correlation is still found in the context of a nonword matching task that does not involve a vocal-articulatory response-demand (Gathercole et al., 1999). Here, two nonwords are presented in succession and the task is to indicate (via keypress) whether or not they are identical or whether two elements (e.g., syllables) have been switched. However, while this finding may rule out an articulatory *production*-based account of the NR-vocabulary correlation, it does not speak to the possible role of (subvocal) articulatory planning in the correlation, because nonword matching span performance could still be supported by such planning despite the absence of a requirement for articulatory output. Indeed, consistent with the notion that it is, we showed in Hughes et al. (2024) that articulatory suppression significantly impairs nonword matching performance, just as it does NR.

In sum, there is evidence that verbal sequence learning, such as that witnessed in word–nonword paired-associate learning and the Hebb repetition effect, and performance in tasks that correlate with verbal sequence learning (NR, nonword matching) is supported to a substantive degree by articulatory planning. There is little convincing evidence that one needs to posit a phonological store in addition to articulatory processes to explain word-form learning, the suggested evolved function of the phonological store. Thus, there is a much simpler solution to the quandary of the evolved function of the phonological store: There is no such quandary because the phenomena ascribed to its action reflect the operation of a system whose evolved function holds little mystery: The planning of coherent (vocal) action (e.g., Fitch, 2018; for a similar view, see Vihman, 2022).

## Summary table

Before closing with some concluding observations, the reader is referred to Table 1 which summarises the key empirical phenomena discussed within the current review, the explanation of, or/and the claims made on the basis of these by both the phonological loop model and the perceptual-motor account, and finally the main pieces of evidence or reasoning that were used to argue in favour of the latter over the former account of each phenomenon.

**Table 1.** Summary of key empirical phenomena, explanations/claims of the phonological loop model, and the perceptual-motor account in relation to these, and main pieces of evidence/reasoning deemed to favour the latter account.

Empirical phenomenon	Phonological loop model explanation/claim	Perceptual-motor account explanation/claim	Key evidence supporting perceptual-motor account over phonological loop model
PSE	Interference between similar item representations during retrieval from a passive abstract-phonological store (e.g., Baddeley et al., 1984)	The effect reflects articulatory sequence planning errors	PSE disappears when articulatory planning is impeded (by AS) regardless of presentation modality (Jones et al., 2004): Pattern of serial-recall errors with “phonologically” similar lists identical to those found in spontaneous speech (Ellis, 1980) and with laboratory-induced “tongue twisters” (Acheson & MacDonald, 2009; Page et al., 2007)
PSE survives AS with auditory but not visual presentation	There exists a passive phonological store to which (only) *auditory–*verbal input has automatic access	Reflects the legacy of passive acoustic-based auditory perceptual-organisation processes	Residual PSE under AS with auditory lists is found primarily at recency (but also throughout list when very short) Residual PSE at recency eliminated by a suffix Residual PSE reinstated if suffix in a different voice from memoranda (despite its phonology remaining unchanged) (Jones et al., 2004, 2006)
ISE	Demonstrates obligatory access of speech or speech-like sound into a phonological store Current view is that changing-state irrelevant sound depletes attentional resources required to form a representation of the order of memoranda used by the phonological store (Page & Norris, 2003)	Reflects a conflict between obligatory acoustic-perceptual processing of the order of changing-state sounds and deliberate sequential articulatory planning of the memoranda	The sound need not be speech (or hence phonological) or, arguably, speech-like to produce an ISE (Jones & Macken, 1993) Engagement in articulatory sequence planning is a precondition for the effect (e.g., Hughes et al., 2007; Jones & Macken, 1993) even with auditory presentation of the memoranda (Jones et al., 2004) Evidence that changing-state irrelevant sound effect is not due to depletion of attentional resources (Hughes, 2014; Hughes et al., 2013)
Nonword repetition and its correlation with vocabulary size	Nonword repetition ability assumed to constitute a particularly pure measure of passive phonological storage capacity (e.g., Baddeley et al., 1998) Correlation between nonword repetition and vocabulary size taken as key evidence for the PS-LLD hypothesis	Nonword repetition performance, like serial recall, largely reflects articulatory-planning ability and so too, therefore, might the correlation between nonword repetition and vocabulary size	Nonword repetition (as well as nonword matching span, which requires no overt vocal response) impaired appreciably when articulatory planning impeded (by AS) (Hughes et al., 2024)
Hebb repetition effect	Provides further evidence for PS-LLD hypothesis Reflects long-term learning within a (non-articulatory) ordering mechanism used by the phonological store (e.g., Hitch et al., 2009; Page et al., 2006)	Primarily reflects the legacy of short-term articulatory planning	Hebb effect impeded/eliminated by articulatory suppression (Sjöblom & Hughes, 2020). This contradicts phonological store-based accounts of the Hebb effect (Hitch et al., 2009; Page et al., 2006) but converges with evidence from other word-form learning settings (e.g., Papagno et al., 1991) Hebb effect impaired by changes across Hebb-list repetitions of the temporal structure of the articulatory plan (Sjöblom & Hughes, 2020)
Typical pattern of performance found in brain-damaged “STM patients”	Such patients have a damaged passive phonological store. Results indicating a possible rehearsal-deficit explained as a secondary, strategic, avoidance of the use of the damaged store (e.g., Vallar & Papagno, 2002)	(Offered currently as a tentative alternative hypothesis): Intact auditory perceptual-organisation-based sequencing of list-items seduces patients into using a defective articulatory-planning system with auditorily but not visually presented lists	Pattern of performance exhibited by the STM patient largely mimics that by neurologically unimpaired participants under articulatory suppression (e.g., Vallar, 2002) Normal spontaneous speech or normal performance in simple tests of speech production does not rule out difficulty with articulatory planning of novel sequences (such as required in serial recall)

PSE: phonological similarity effect; ISE: irrelevant speech/sound effect; AS: articulatory suppression; PS-LLD: phonological store as a language-learning device.

## Concluding observations: the perceptual-motor approach as an emergent-property approach

The influence of the phonological loop model on the perceptual-motor approach cannot be overstated; indeed, the perceptual-motor approach might never have emerged without it. The main reason for this is the emphasis placed in both approaches on the role of articulatory processes although, as discussed, the function of such processes is quite distinct in the two views. The perceptual-motor approach is also, ultimately, more conceptually similar to other approaches that deny the need to posit a distinct STM system and see STM performance instead as an emergent byproduct of the action of other processes (e.g., Acheson & MacDonald, 2009; Cowan, 2019; Craik & Lockhart, 1972; Crowder, 1993; MacDonald, 2016). These accounts embody the idea that “short-term memory” is little more than the activated portion of LTM (e.g., Cowan, 1999, 2019; Ruchkin et al., 2003). A specific instantiation of this approach is the language-based view in which verbal STM reflects the transient activation of the same representations that are used to comprehend and produce language (e.g., Acheson & MacDonald, 2009).

It is clear, however, that activated LTM is not sufficient on its own as an account of serial STM task performance because, as discussed earlier, the quintessential feature of such a task is that it is about dealing with *novelty* (specifically, a novel sequence), that is, it is precisely about dealing with something that is *not* already represented in LTM (e.g., Norris, 2017). In recognising the need to assume some form of novel processing in the context of an STM task, Cowan (2019) suggests that such processing might take the form of “rapid learning” within the LTM system, whereby new associations within a presented stimulus-set are formed. However, it is unclear in Cowan (2019) what the rapid learning mechanism might be. And while it is generally accepted that LTM is the system that represents the *products* of learning, it is unclear how an LTM system, on its own, could add new information to itself (i.e., learn). Thus, something in addition to LTM is required to carry out the “rapid learning.” That something, according to the Working Memory model, is a short-term store (or set of stores for different kinds of input) and. on the perceptual-motor approach, that something is an articulatory plan or/and the products of obligatory auditory streaming. Interestingly, Norris’s (2017) strident reassertion of the need for short-term processes separate from LTM (with which I agree) is coupled with a distinct ambivalence regarding the core nature of the phonological store: “A short-term phonological store. . .might be a specifically mnemonic store, *or parasitic on processes responsible for perception or speech production*” (Norris, 2017, p. 1003, emphasis added). I would suggest, however, that if the phonological store is indeed parasitic on perception or/and speech production, it is not a phonological store at all, at least not as originally or most frequently conceived (Baddeley, 1986, 2007).

Some authors have recently begun, therefore, to incorporate the central features of the perceptual-motor approach into the STM-as-activated-LTM approach:

One could . . . consider activated long-term memories to include fleeting representations temporarily preserved by perceptual systems and information kept active by motor re-instantiation. Sensory-motor recruitment makes it unnecessary to impose dedicated, specialized short-term “slave” systems into the embedded process framework’s activated memories: The activation of perceptual and motor systems can serve the memory system without creating redundancy. (Morey et al., 2019, p. 158)

I contend, however, that the motor system—especially when passive auditory perceptual organisation cannot play a role (e.g., with visual presentation)—does much more than merely “re-instantiate” representations produced via perceptual systems: It *generates* the temporarily extended object that will form the basis of the reproduction of the presented sequence. It is also then the generation (and repeated generation) of a new motor-object that supports the entry of the initially novel input into the LTM system (as witnessed, e.g., in Hebb sequence learning; Sjöblom & Hughes, 2020). Moreover, as also argued by Norris (2017, 2019), once it is conceded that there are distinct STM mechanisms, the STM-as-activated-LTM view loses much of its force. Thus, the key claim of the perceptual-motor account is that the item and sub-item representations that support performance in STM tasks are activated LTM representations—it is unclear how they could be anything else (see also Norris, 2017)—but motor processes or/and perceptual-organisational processes act upon those activated representations and it is those processes that subserve both immediate performance but also lead to the formation of new long-term representations. Thus, the perceptual-motor account heeds Hebb’s (1958) early warning to resist the temptation to explain the “holding problem” as he termed it (or what might be called the “short-term storage problem”) with a different mechanism from that responsible for immediate stimulus–response behaviours, that is, some sort of “container” that has the very capability that needs to be explained (Buchsbaum & D’Esposito, 2019; Hebb, 1958; Macken & Jones, 2003).

It was stated recently that one of the major questions for the Working Memory model that remains unanswered is “how does the operation of the phonological loop link to theories of speech perception and production?” (Baddeley et al., 2021, p. 14). I argue that that there is no need to specify such a link: A full understanding of speech (and more generally, auditory) perception, speech planning, and production, and the ways in which these processes interact with one another and with extant knowledge—while still a good way off—will provide a full understanding of verbal serial STM performance and verbal sequence learning, without a need to invoke a separate short-term (phonological) store.

## References

[bibr1-17470218241257885] Acheson D. J. MacDonald M. C. (2009a). Twisting tongues and memories: Explorations of the relationship between language production and verbal working memory. Journal of Memory and Language, 60, 329–350. 10.1016/j.jml.2008.12.002 21165150 PMC3001594

[bibr2-17470218241257885] Acheson D. J. MacDonald M. C. (2009b). Verbal working memory and language production: Common approaches to the serial ordering of verbal information. Psychological Bulletin, 135(1), 50–68. 19210053 10.1037/a0014411 PMC3000524

[bibr3-17470218241257885] Archibald L. M. D. Gathercole S. E. Joanisse M. F. (2009). Multisyllabic nonwords: More than a string of syllables. The Journal of the Acoustical Society of America, 125(3), 1712–1722. 10.1121/1.3076200 19275328

[bibr4-17470218241257885] Baddeley A. D. (1966). Short-term memory for word sequences as a function of acoustic, semantic and formal similarity. Quarterly Journal of Experimental Psychology, 18(4), 362–365. 10.1080/14640746608400055 5956080

[bibr5-17470218241257885] Baddeley A. D. (1976). The psychology of memory. Basic Books.

[bibr6-17470218241257885] Baddeley A. D. (1986). Working memory. Oxford University Press.

[bibr7-17470218241257885] Baddeley A. D. (2000). The phonological loop and the irrelevant speech effect: Some comments on Neath (2000). Psychonomic Bulletin & Review, 7(3), 544–549. 11082863 10.3758/bf03214369

[bibr8-17470218241257885] Baddeley A. D. (2003). Working memory: Looking back and looking forward. Nature Reviews Neuroscience, 4(10), 829–839. 14523382 10.1038/nrn1201

[bibr9-17470218241257885] Baddeley A. D. (2007). Working memory, thought and action. Oxford.

[bibr10-17470218241257885] Baddeley A. D. (2012). Working memory: Theories, models, and controversies. Annual Review of Psychology, 63, 1–29. 10.1146/annurev-psych-120710-100422 21961947

[bibr11-17470218241257885] Baddeley A. D. (2021). Developing the concept of working memory: The role of neuropsychology. Archives of Clinical Neuropsychology, 36(6), 861–873. 34312672 10.1093/arclin/acab060 PMC11979773

[bibr12-17470218241257885] Baddeley A. D. Gathercole S. Papagno C. (1998). The phonological loop as a language learning device. Psychological Review, 105(1), 158–173. 9450375 10.1037/0033-295x.105.1.158

[bibr13-17470218241257885] Baddeley A. D. Hitch G. J. (1974). Working memory. In Bower G. (Ed.), The psychology of learning and motivation (Vol. 8, pp. 47–90). Academic Press.

[bibr14-17470218241257885] Baddeley A. D. Hitch G. J. (2019). The phonological loop as a buffer store: An update. Cortex, 112, 91–106. 29941299 10.1016/j.cortex.2018.05.015

[bibr15-17470218241257885] Baddeley A. D. Hitch G. J. Allen R. J. (2021). A multicomponent model of working memory. In Logie R. H. Camos V. Cowan N. (Eds.), Working memory: State of the science (pp. 10–43). Oxford University Press.

[bibr16-17470218241257885] Baddeley A. D. Larsen J. D. (2007). The phonological loop unmasked? A comment on the evidence for a “perceptual-gestural” alternative. The Quarterly Journal of Experimental Psychology, 60(4), 497–504. 17455059 10.1080/17470210601147572

[bibr17-17470218241257885] Baddeley A. D. Lewis V. (1984). When does rapid presentation enhance digit span? Bulletin of the Psychonomic Society, 22, 403–405.

[bibr18-17470218241257885] Baddeley A. D. Lewis V. Vallar G. (1984). Exploring the articulatory loop. Quarterly Journal of Experimental Psychology, A36, 233–252.

[bibr19-17470218241257885] Baddeley A. Papagno C. Vallar G. (1988). When long-term learning depends on short-term storage. Journal of Memory and Language, 27(5), 586–595.

[bibr20-17470218241257885] Baddeley A. D. Thomson N. Buchanan M. (1975). Word length and the structure of short-term memory. Journal of Verbal Learning and Verbal Behavior, 14(6), 575–589.

[bibr21-17470218241257885] Baddeley A. D. Vallar G. Wilson B. (1987). Sentence comprehension and phonological memory: Some neuropsychological evidence. In Coltheart M. (Ed.), Attention and performance XII. The psychology of reading (pp. 509–529). Erlbaum.

[bibr22-17470218241257885] Baddeley A. D. Wilson B. A. (1988). Comprehension and working memory: A single case neuropsychological study. Journal of Memory and Language, 27, 479–498.

[bibr23-17470218241257885] Basso A. Spinndler H. Vallar G. Zanobio M. E. (1982). Left hemisphere damage and selective impairment of auditory-verbal short-term memory. Neuropsychologica, 20, 263–274. 10.1016/0028-3932(82)90101-4 7121794

[bibr24-17470218241257885] Beaman C. P. Jones D. M. (1997). Role of serial order in the irrelevant speech effect: Tests of the changing state hypothesis. Journal of Experimental Psychology: Learning, Memory & Cognition, 23, 459–471.

[bibr25-17470218241257885] Beaman C. P. Jones D. M. (1998). Irrelevant sound disrupts order information in free recall as in serial recall. The Quarterly Journal of Experimental Psychology: Section A, 51(3), 615–636. 10.1080/713755774 9745380

[bibr26-17470218241257885] Bell R. Röer J. P. Lang A. G. Buchner A. (2019). Distraction by steady-state sounds: Evidence for a graded attentional model of auditory distraction. Journal of Experimental Psychology: Human Perception and Performance, 45(4), 500–512. 30816785 10.1037/xhp0000623

[bibr27-17470218241257885] Berndt R. S. Mitchum C. C. Price T. R. (1991). Short-term memory and sentence comprehension: An investigation of a patient with crossed aphasia. Brain, 114(1), 263–280. 1998886

[bibr28-17470218241257885] Bhatarah P. Ward G. Smith J. Hayes L. (2009). Examining the relationship between free recall and immediate serial recall: Similar patterns of rehearsal and similar effects of word length, presentation rate, and articulatory suppression. Memory & Cognition, 37, 689–713. 19487760 10.3758/MC.37.5.689

[bibr29-17470218241257885] Binkofski F. Buccino G. Zilles K. Fink G. R. (2004). Supramodal representation of objects and actions in the human inferior temporal and ventral premotor cortex. Cortex, 40, 159–161. 15174449 10.1016/s0010-9452(08)70933-x

[bibr30-17470218241257885] Bregman A. S. (1990). Auditory scene analysis. MIT Press.

[bibr31-17470218241257885] Bregman A. S. Campbell J. (1971). Primary auditory stream segregation and perception of order in rapid sequences of tones. Journal of Experimental Psychology, 89, 244–249. 5567132 10.1037/h0031163

[bibr32-17470218241257885] Bridges A. M. Jones D. M. (1996). Word dose in the disruption of serial recall by irrelevant speech: Phonological confusions or changing state? The Quarterly Journal of Experimental Psychology: Section A, 49(4), 919–939.

[bibr33-17470218241257885] Buchsbaum B. R. D’Esposito M. (2008). The search for the phonological store: From loop to convolution. Journal of Cognitive Neuroscience, 20, 762–778. 18201133 10.1162/jocn.2008.20501

[bibr34-17470218241257885] Buchsbaum B. R. D’Esposito M. (2008). The search for the phonological store: From loop to convolution. Journal of Cognitive Neuroscience, 20, 762–778. 18201133 10.1162/jocn.2008.20501

[bibr35-17470218241257885] Buchsbaum B. R. D’Esposito M. (2019). A sensorimotor view of verbal working memory. Cortex, 112, 134–148. 30639088 10.1016/j.cortex.2018.11.010

[bibr36-17470218241257885] Burgess N. Hitch G. J. (2006). A revised model of short-term memory and long-term learning of verbal sequences. Journal of Memory and Language, 55(4), 627–652.

[bibr37-17470218241257885] Burgess N. Hitch G. (2005). Computational models of working memory: putting long-term memory into context. Trends in Cognitive Sciences, 9(11), 535–541. 16213782 10.1016/j.tics.2005.09.011

[bibr38-17470218241257885] Caplan D. Vanier M. Backer C. (1986). A case study of reproduction conduction aphasia I: Word production. Cognitive Neuropsychology, 3, 99–128.

[bibr39-17470218241257885] Caplan D. Waters G. Howard D. (2012). Slave systems in verbal short-term memory. Aphasiology, 26(3–4), 279–316. 10.1080/02687038.2011.642795 PMC3859463 24347786

[bibr40-17470218241257885] Catani M. Jones D. K. Ffytche D. H. (2005). Perisylvian language networks of the human brain. Annals of Neurology, 57(1), 8–16. 15597383 10.1002/ana.20319

[bibr41-17470218241257885] Chomsky N. Halle M. (1968). The sound pattern of English. Harper & Row.

[bibr42-17470218241257885] Colle H. A. Welsh A. (1976). Acoustic masking in primary memory. Journal of Verbal Learning and Verbal Behavior, 15, 17–32.

[bibr43-17470218241257885] Conrad R. (1964). Acoustic confusions in immediate memory. British Journal of Psychology, 55, 75–84.

[bibr44-17470218241257885] Conrad R. Hull A. J. (1964). Information, acoustic confusion and memory span. British Journal of Psychology, 55, 429–432. 14237884 10.1111/j.2044-8295.1964.tb00928.x

[bibr45-17470218241257885] Cowan N. (1999). An embedded-processes model of working memory. Models of Working Memory: Mechanisms of Active Maintenance and Executive Control, 20(506), 1013–1019.

[bibr46-17470218241257885] Cowan N. (2019). Short-term memory based on activated long-term memory: A review in response to Norris (2017). Psychological Bulletin, 145(8), 822–847. 31328941 10.1037/bul0000199 PMC6650160

[bibr47-17470218241257885] Craik F. I. Levy B. A. (1970). Semantic and acoustic information in primary memory. Journal of Experimental Psychology, 86(1), 77–82.

[bibr48-17470218241257885] Craik F. I. Lockhart R. S. (1972). Levels of processing: A framework for memory research. Journal of Verbal Learning and Verbal Behavior, 11(6), 671–684.

[bibr49-17470218241257885] Crowder R. G. (1971). The sound of vowels and consonants in immediate memory. Journal of Verbal Learning and Verbal Behavior, 10, 587–596.

[bibr50-17470218241257885] Crowder R. G. (1978). Mechanisms of backward masking in the stimulus suffix effect. Psychological Review, 85, 502–524. 734019

[bibr51-17470218241257885] Crowder R. G. (1993). Short-term memory: Where do we stand? Memory & Cognition, 21, 142–145. 8469121 10.3758/bf03202725

[bibr52-17470218241257885] Darwin C. J. Baddeley A. D. (1974). Acoustic memory and the perception of speech. Cognitive Psychology, 6, 41–60.

[bibr53-17470218241257885] Devlin J. T. Matthews P. M. Rushworth M. F. (2003). Semantic processing in the left inferior prefrontal cortex: A combined functional magnetic resonance imaging and transcranial magnetic stimulation study. Journal of Cognitive Neuroscience, 15(1), 71–84. 12590844 10.1162/089892903321107837

[bibr54-17470218241257885] Ellis A. W. (1980). Errors in speech and short-term memory: The effects of phonemic similarity and syllable position. Journal of Verbal Learning and Verbal Behavior, 19, 624–634.

[bibr55-17470218241257885] Ellis N. C. Hennelly R. A. (1980). A bilingual word-length effect: Implications for intelligence testing and the relative ease of mental calculation in Welsh and English. British Journal of Psychology, 71(1), 43–51.

[bibr56-17470218241257885] Fitch W. T. (2018). The biology and evolution of speech: A comparative analysis. Annual Review of Linguistics, 4, 255–279.

[bibr57-17470218241257885] Fournet N. Juphard A. Monnier C. Roulin J. L. (2003). Phonological similarity in free and serial recall: The effect of increasing retention intervals. International Journal of Psychology, 38, 384–389. 10.1080/00207590344000204

[bibr58-17470218241257885] Frankish C. (1985). Modality-specific grouping effects in short-term memory. Journal of Memory and Language, 24(2), 200–209.

[bibr59-17470218241257885] Frankish C. (1989). Perceptual organization and precategorical acoustic storage. Journal of Experimental Psychology: Learning, Memory, and Cognition, 15(3), 469–479. 2524546 10.1037//0278-7393.15.3.469

[bibr60-17470218241257885] Gathercole S. E. (1995). Is nonword repetition a test of phonological memory or long-term knowledge? It all depends on the nonwords. Memory & Cognition, 23(1), 83–94. 10.3758/BF03210559 7885268

[bibr61-17470218241257885] Gathercole S. E. (2006). Nonword repetition and word learning: The nature of the relationship. Applied Psycholinguistics, 27, 513–543.

[bibr62-17470218241257885] Gathercole S. E. Baddeley A. D. (1989). Evaluation of the role of phonological STM in the development of vocabulary in children: A longitudinal study. Journal of Memory and Language, 28, 200–213.

[bibr63-17470218241257885] Gathercole S. E. Baddeley A. D. (1993). Working memory and language. Erlbaum.

[bibr64-17470218241257885] Gathercole S. E. Service E. Hitch G. J. Adams A.-M. Martin A. J. (1999). Phonological short-term memory and vocabulary development: Further evidence on the nature of the relationship. Applied Cognitive Psychology, 13, 65–77.

[bibr65-17470218241257885] Glanzer M. Koppenaal L. Nelson R. (1972). Effects of relations between words on short-term storage and long-term storage. Journal of Verbal Learning and Verbal Behavior, 11(4), 403–416.

[bibr66-17470218241257885] Goldstein M. (1968). Some slips of the tongue. Psychological Reports, 22, 1009–1013. 5653350 10.2466/pr0.1968.22.3.1009

[bibr67-17470218241257885] Grush R. (2004). The emulation theory of representation: Motor control, imagery, and perception. Behavioral and Brain Sciences, 27(3), 377–396. 15736871 10.1017/s0140525x04000093

[bibr68-17470218241257885] Gupta P. (2005). Primacy and recency in nonword repetition. Memory, 13(3-4 ), 318–324. 15948616 10.1080/09658210344000350

[bibr69-17470218241257885] Gupta P. Lipinski J. Abbs B. Lin P. H. (2005). Serial position effects in nonword repetition. Journal of Memory and Language, 53(1), 141–162.

[bibr70-17470218241257885] Hanley J. R. (1997). Does articulatory suppression remove the irrelevant speech effect? Memory, 5, 423–431. 9231151 10.1080/741941394

[bibr71-17470218241257885] Hanley J. R. Bakopoulou E. (2003). Irrelevant speech, articulatory suppression and phonological similarity: A test of the phonological loop model and the feature model. Psychonomic Bulletin & Review, 10, 435–444. 12921421 10.3758/bf03196503

[bibr72-17470218241257885] Hanley J. R. Bourgaize J. (2018). Similarities between the irrelevant sound effect and the suffix effect. Memory & Cognition, 46, 841–848. 29600481 10.3758/s13421-018-0806-8

[bibr73-17470218241257885] Hanley J. R. Broadbent C. (1987). The effects of unattended speech on serial recall following auditory presentation. British Journal of Psychology, 78, 287–297.

[bibr74-17470218241257885] Hanley J. R. Hayes A. (2012). The irrelevant sound effect under articulatory suppression: Is it a suffix effect? Journal of Experimental Psychology: Learning, Memory and Cognition, 38, 482–487. 21928934 10.1037/a0025600

[bibr75-17470218241257885] Hanley J. R. Shah N. (2012). The irrelevant sound effect under articulatory suppression is a suffix effect even with 5-item lists. Memory, 20, 482–487. 10.1080/09658211.2012.670249 22497740

[bibr76-17470218241257885] Hardcastle W. Hewlett N. (2006). Coarticulation: Theory, data, and techniques. Cambridge University Press.

[bibr77-17470218241257885] Hebb D. O. (1958). A textbook of psychology. Saunders.

[bibr78-17470218241257885] Hebb D. O. (1961). Distinctive features of learning in the higher animal. In Delafresnaye J. F. (Ed.), Brain mechanisms and learning (pp. 37–46). Oxford University Press.

[bibr79-17470218241257885] Henson R. N. A. (1998). Short-term memory for serial order: The Start-End model. Cognitive Psychology, 36, 73–137. 9721198 10.1006/cogp.1998.0685

[bibr80-17470218241257885] Hintzman D. L. (1965). Classification and aural coding in short-term memory. Psychonomic Science, 3, 161–162.

[bibr81-17470218241257885] Hintzman D. L. (1967). Articulatory coding in short-term memory. Journal of Verbal Learning and Verbal Behavior, 6, 312–316.

[bibr82-17470218241257885] Hitch G. J. Burgess N. Towse J. N. Culpin V. (1996). Temporal grouping effects in immediate recall: A working memory analysis. The Quarterly Journal of Experimental Psychology Section A, 49(1), 116–139.

[bibr83-17470218241257885] Hitch G. J. Flude B. Burgess N. (2009). Slave to the rhythm: Experimental tests of a model for verbal short-term memory and long-term sequence learning. Journal of Memory and Language, 61(1), 97–111.

[bibr84-17470218241257885] Hughes R. W. (2014). Auditory distraction: A duplex-mechanism account. PsyCh Journal, 3, 30–41. 26271638 10.1002/pchj.44

[bibr85-17470218241257885] Hughes R. W. Chamberland C. Tremblay S. Jones D. M. (2016). Perceptual-motor determinants of auditory-verbal serial short-term memory. Journal of Memory and Language, 90, 126–146.

[bibr86-17470218241257885] Hughes R. W. Harvey H. Mills J. L. (2024). Nonword repetition: An articulatory approach [Manuscript in preparation].

[bibr87-17470218241257885] Hughes R. W. Hurlstone M. J. Marsh J. E. Vachon F. Jones D. M. (2013). Cognitive control of auditory distraction: Impact of task difficulty, foreknowledge, and working memory capacity supports duplex-mechanism account. Journal of Experimental Psychology: Human Perception and Performance, 39, 539–553. 10.1037/a0029064 22731996

[bibr88-17470218241257885] Hughes R. W. Jones D. M. (2001). The intrusiveness of sound: Laboratory findings and their implications for noise abatement. Noise & Health, 13, 55–74. 12678935

[bibr89-17470218241257885] Hughes R. W. Jones D. M. (2005). The impact of order incongruence between a task-irrelevant auditory sequence and a task-relevant visual sequence. Journal of Experimental Psychology: Human Perception and Performance, 31, 316–327. 15826233 10.1037/0096-1523.31.2.316

[bibr90-17470218241257885] Hughes R. W. Marsh J. E. (2017). The functional determinants of short-term memory: Evidence from perceptual-motor interference in verbal serial recall. Journal of Experimental Psychology: Learning, Memory, and Cognition, 43, 537–551. 27668482 10.1037/xlm0000325

[bibr91-17470218241257885] Hughes R. W. Marsh J. E. (2019). Dissociating two forms of auditory distraction in a novel Stroop serial recall experiment. Auditory Perception & Cognition, 2(3), 129–142.

[bibr92-17470218241257885] Hughes R. W. Marsh J. E. (2020). When is forewarned forearmed? Predicting auditory distraction in short-term memory. Journal of Experimental Psychology: Learning, Memory, and Cognition, 46(3), 427–442. 10.1037/xlm0000736 31180705

[bibr93-17470218241257885] Hughes R. W. Marsh J. E. Jones D. M. (2009). Perceptual–gestural (mis)mapping in serial short-term memory: The impact of talker variability. Journal of Experimental Psychology: Learning, Memory, and Cognition, 35(6), 1411–1425. 19857013 10.1037/a0017008

[bibr94-17470218241257885] Hughes R. W. Marsh J. E. Jones D. M. (2011). Role of serial order in the impact of talker variability on short-term memory: Testing a perceptual organization-based account. Memory & Cognition, 39(8), 1435–1447. 21638105 10.3758/s13421-011-0116-x

[bibr95-17470218241257885] Hughes R. W. Vachon F. Jones D. M. (2005). Auditory attentional capture during serial recall: Violations at encoding of an algorithm-based neural model? Journal of Experimental Psychology: Learning, Memory & Cognition, 31, 736–749. 16060777 10.1037/0278-7393.31.4.736

[bibr96-17470218241257885] Hughes R. W. Vachon F. Jones D. M. (2007). Disruption of short-term memory by changing and deviant sounds: Support for a duplex-mechanism account of auditory distraction. Journal of Experimental Psychology: Learning, Memory, & Cognition, 33, 1050–1061. 17983312 10.1037/0278-7393.33.6.1050

[bibr97-17470218241257885] Hurlstone M. J. Hitch G. J. Baddeley A. D. (2014). Memory for serial order across domains: An overview of the literature and directions for future research. Psychological Bulletin, 140(2), 1–35. 24079725 10.1037/a0034221

[bibr98-17470218241257885] Jobard G. Crivello F. Tzourio-Mazoyer N. (2003). Evaluation of the dual route theory of reading: A metanalysis of 35 neuroimaging studies. NeuroImage, 20(2), 693–712. 14568445 10.1016/S1053-8119(03)00343-4

[bibr99-17470218241257885] Jones D. M. Alford D. Bridges A. Tremblay S. Macken W. J. (1999). Organizational factors in selective attention: The interplay of acoustic distinctiveness and auditory streaming in the irrelevant sound effect. Journal of Experimental Psychology: Learning, Memory, and Cognition, 25, 464–473.

[bibr100-17470218241257885] Jones D. M. Hughes R. W. Macken W. J. (2006). Perceptual organization masquerading as phonological storage: Further support for a perceptual-gestural view of short-term memory. Journal of Memory and Language, 54(2), 265–281.

[bibr101-17470218241257885] Jones D. M. Hughes R. W. Macken W. J. (2007). The phonological store abandoned. The Quarterly Journal of Experimental Psychology, 60(4), 505–511. 17455060 10.1080/17470210601147598

[bibr102-17470218241257885] Jones D. M. Macken W. J. (1993). Irrelevant tones produce an irrelevant speech effect: Implications for phonological coding in working memory. Journal of Experimental Psychology: Learning Memory & Cognition, 19, 369–381.

[bibr103-17470218241257885] Jones D. M. Macken W. J. (1995a). Organizational factors in the effect of irrelevant speech: The role of spatial location and timing. Memory & Cognition, 23, 192–200. 10.3758/BF03197221 7731364

[bibr104-17470218241257885] Jones D. M. Macken W. J. (1995b). Phonological similarity in the irrelevant speech effect: Within-or between-stream similarity? Journal of Experimental Psychology: Learning, Memory, and Cognition, 21, 103–115.

[bibr105-17470218241257885] Jones D. M. Macken W. J. Murray A. C. (1993). Disruption of visual short-term memory by changing-state auditory stimuli: The role of segmentation. Memory & Cognition, 21, 318–328. 8316094 10.3758/bf03208264

[bibr106-17470218241257885] Jones D. M. Macken W. J. Nicholls A. P. (2004). The phonological store of working memory: Is it phonological and is it a store? Journal of Experimental Psychology: Learning Memory & Cognition, 30, 656–674. 15099134 10.1037/0278-7393.30.3.656

[bibr107-17470218241257885] Jones D. M. Madden C. Miles C. (1992). Privileged access by irrelevant speech to short-term memory: The role of changing state. Quarterly Journal of Experimental Psychology Section A, 44(4), 645–669. 10.1080/14640749208401304 1615168

[bibr108-17470218241257885] Jones D. M. Tremblay S. (2000). Interference by process or content? A reply to Neath (2000). Psychonomic Bulletin & Review, 7, 550–558. 11082864 10.3758/bf03214370

[bibr109-17470218241257885] Kinsbourne M. (1972). Behavioral analysis of the repetition deficit in conduction aphasia. Neurology, 22, 1126–1132. 4673556 10.1212/wnl.22.11.1126

[bibr110-17470218241257885] Klatte M. Lee N. Hellbruck J. (2002). Effects of irrelevant speech and articulatory suppression on serial recall of heard and read materials. Psychologische Beitrage, 44, 166–186.

[bibr111-17470218241257885] Koffka K. (1935). Principles of gestalt psychology. Harcourt, Brace.

[bibr112-17470218241257885] Lackner J. R. Goldstein L. M. (1974). Primary auditory stream segregation of repeated consonant—vowel sequences. The Journal of the Acoustical Society of America, 56(5), 1651–1652. 4427037 10.1121/1.1903493

[bibr113-17470218241257885] Larsen J. D. Baddeley A. D. (2003). Disruption of verbal STM by irrelevant speech, articulatory suppression, and manual tapping: Do they have a common source? Quarterly Journal of Experimental Psychology Section A, 56(8), 1249–1268. 10.1080/02724980244000765 14578082

[bibr114-17470218241257885] Larsen J. D. Baddeley A. D. Andrade J. (2000). Phonological similarity and the irrelevant speech effect: Implications for models of short-term verbal memory. Memory, 8, 145–157. 10889899 10.1080/096582100387579

[bibr115-17470218241257885] LeCompte D. C. (1996). Irrelevant speech, serial rehearsal, and temporal distinctiveness: A new approach to the irrelevant speech effect. Journal of Experimental Psychology: Learning, Memory, and Cognition, 22, 1154–1165. 8805820 10.1037//0278-7393.22.5.1154

[bibr116-17470218241257885] LeCompte D. C. Shaibe D. M. (1997). On the irrelevance of phonological similarity to the irrelevant speech effect. Quarterly Journal of Experimental Psychology: Human Experimental Psychology, 50(A), 100–118. 10.1080/713755679 9080790

[bibr117-17470218241257885] Levy B. (1971). Role of articulation in auditory and visual short-term memory. Journal of Verbal Learning and Verbal Behaviour, 10, 123–132.

[bibr118-17470218241257885] Levy B. A. Murdock B. B. Jr. (1968). The effects of delayed auditory feedback and intralist similarity in short-term memory. Journal of Verbal Learning and Verbal Behavior, 7(5), 887–894.

[bibr119-17470218241257885] MacDonald M. C. (2016). Speak, act, remember: The language-production basis of serial order and maintenance in verbal memory. Current Directions in Psychological Science, 25, 47–53. 10.1177/0963721415620776

[bibr120-17470218241257885] MacKay D. G. (1970). Spoonerisms: The structure of errors in the serial order of speech. Neuropsychologia, 8(3), 323–350. 5522566 10.1016/0028-3932(70)90078-3

[bibr121-17470218241257885] Macken B. Taylor J. C. Jones D. M. (2014). Language and short-term memory: The role of perceptual-motor affordance. Journal of Experimental Psychology: Learning, Memory, and Cognition, 40(5), 1257–1270. 24797440 10.1037/a0036845 PMC4143182

[bibr122-17470218241257885] Macken W. J. Jones D. M. (2003). Reification of phonological storage. Quarterly Journal of Experimental Psychology, 56A, 1279–1288. 10.1080/02724980245000052 14578084

[bibr123-17470218241257885] Macken W. J. Tremblay S. Houghton R. J. Nicholls A. P. Jones D. M. (2003). Does auditory streaming require attention? Evidence from attentional selectivity in short-term memory. Journal of Experimental Psychology: Human Perception and Performance, 29(1), 43–51. 12669746 10.1037//0096-1523.29.1.43

[bibr124-17470218241257885] MacNeilage P. F. (1998). The frame/content theory of evolution of speech Production. Behavioral and Brain Sciences, 21(4), 499–511. 10097020 10.1017/s0140525x98001265

[bibr125-17470218241257885] Maidment D. W. Macken W. J. (2012). The ineluctable modality of the audible: Perceptual determinants of auditory verbal short-term memory. Journal of Experimental Psychology: Human Perception and Performance, 38(4), 989–997. 22468724 10.1037/a0027884

[bibr126-17470218241257885] Maybery M. T. Parmentier F. B. Jones D. M. (2002). Grouping of list items reflected in the timing of recall: Implications for models of serial verbal memory. Journal of Memory and Language, 47(3), 360–385.

[bibr127-17470218241257885] Melby-Lervåg M. Lervåg A. Lyster S. A. H. Klem M. Hagtvet B. Hulme C. (2012). Nonword-repetition ability does not appear to be a causal influence on children’s vocabulary development. Psychological Science, 23(10), 1092–1098. 22923338 10.1177/0956797612443833

[bibr128-17470218241257885] Miles C. Jones D. M. Madden C. A. (1991). Locus of the irrelevant speech effect in short-term memory. Journal of Experimental Psychology: Learning, Memory, and Cognition, 17(3), 578–584.

[bibr129-17470218241257885] Miller G. A. Chomsky N. (1963). Finitary models of language users. In Luce R. D. Bush R. R. Galanter E. (Eds.), Handbook of mathematical psychology (Vol. 2, pp. 419–492). Wiley.

[bibr130-17470218241257885] Morey C. C. Rhodes S. Cowan N. (2019). Sensory-motor integration and brain lesions: Progress toward explaining domain-specific phenomena within domain-general working memory. Cortex, 112, 149–161. 30612701 10.1016/j.cortex.2018.11.030

[bibr131-17470218241257885] Murray A. Jones D. M. (2002). Articulatory complexity at item boundaries in serial recall: The case of Welsh and English digit span. Journal of Experimental Psychology: Learning, Memory, and Cognition, 28(3), 594–598. 10.1037/0278-7393.28.3.594 12018511

[bibr132-17470218241257885] Murray D. J. (1968). Articulation and acoustic confusability in short-term memory. Journal of Experimental Psychology, 78, 679–684.

[bibr133-17470218241257885] Nairne J. S. (2002). Remembering over the short-term: The case against the standard model. Annual Review of Psychology, 53(1), 53–81. 10.1146/annurev.psych.53.100901.135131 11752479

[bibr134-17470218241257885] Neath I. (2000). Modeling the effects of irrelevant speech on memory. Psychonomic Bulletin & Review, 7, 403–423. 11082850 10.3758/bf03214356

[bibr135-17470218241257885] Neath I. Nairne J. S. (1995). Word-length effects in immediate memory: Overwriting trace decay theory. Psychonomic Bulletin & Review, 2, 429–441. 24203783 10.3758/BF03210981

[bibr136-17470218241257885] Newell A. Rosenbloom P. S. (1981). Mechanisms of skill acquisition and the law of practice. Cognitive Skills and Their Acquisition, 1, 1–55.

[bibr137-17470218241257885] Nicholls A. P. Jones D. M. (2002). Capturing the suffix: Cognitive streaming in immediate serial recall. Journal of Experimental Psychology: Learning, Memory and Cognition, 28, 12–28. 11827075 10.1037//0278-7393.28.1.12

[bibr138-17470218241257885] Nimmo L. M. Roodenrys S. (2006). The influence of phoneme position overlap on the phonemic similarity effect in nonword recall. Quarterly Journal of Experimental Psychology, 59(3), 577–596. 10.1080/02724980443000845 16627357

[bibr139-17470218241257885] Norris D. (2017). Short-term memory and long-term memory are still different. Psychological Bulletin, 143, 992–1009. 28530428 10.1037/bul0000108 PMC5578362

[bibr140-17470218241257885] Norris D. Page M. Hall J. (2018). Learning nonwords: The Hebb repetition effect as a model of word learning. Memory, 26, 852–857. 10.1080/09658211.2017.1416639 29297757 PMC5954922

[bibr141-17470218241257885] Page M. P. A. Cumming N. Norris D. Hitch G. J. McNeil A. M. (2006). Repetition learning in the immediate serial recall of visual and auditory materials. Journal of Experimental Psychology: Learning, Memory and Cognition, 32(4), 716–733. 16822143 10.1037/0278-7393.32.4.716

[bibr142-17470218241257885] Page M. P. Madge A. Cumming N. Norris D. G. (2007). Speech errors and the phonological similarity effect in short-term memory: Evidence suggesting a common locus. Journal of Memory and Language, 56(1), 49–64.

[bibr143-17470218241257885] Page M. P. A. Norris D. (1998). The primacy model: A new model of immediate serial recall. Psychological Review, 105, 761–781. 9830378 10.1037/0033-295x.105.4.761-781

[bibr144-17470218241257885] Page M. P. A. Norris D. (2003). The irrelevant sound effect: What needs modelling, and a tentative model. Quarterly Journal of Experimental Psychology, A, 56, 1289–1300. 10.1080/02724980343000233 14578085

[bibr145-17470218241257885] Page M. P. A. Norris D. (2009). A model linking immediate serial recall, the Hebb repetition effect and the learning of phonological word forms. Philosophical Transactions of the Royal Society of London B: Biological Sciences, 364(1536), 3737–3753. 19933143 10.1098/rstb.2009.0173 PMC2846317

[bibr146-17470218241257885] Papagno C. Valentine T. Baddeley A. (1991). Phonological short-term memory and foreign-language vocabulary learning. Journal of Memory and Language, 30(3), 331–347.

[bibr147-17470218241257885] Papagno C. Vallar G. (1992). Phonological short-term memory and the learning of novel words: The effect of phonological similarity and item length. The Quarterly Journal of Experimental Psychology Section A, 44(1), 47–67.

[bibr148-17470218241257885] Papagno C. Vallar G. (1995). Verbal short-term memory and vocabulary learning in polyglots. The Quarterly Journal of Experimental Psychology Section A, 48(1), 98–107. 10.1080/14640749508401378 7754088

[bibr149-17470218241257885] Pattamadilok C. Knierim I. N. Duncan K. J. K. Devlin J. T. (2010). How does learning to read affect speech perception? Journal of Neuroscience, 30(25), 8435–8444. 20573891 10.1523/JNEUROSCI.5791-09.2010 PMC6634630

[bibr150-17470218241257885] Petersen S. E. Fox P. T. Posner M. I. Mintun M. Raichle M. E. (1988). Positron emission tomographic studies of the cortical anatomy of single-word processing. Nature, 331(6157), 585–589. 3277066 10.1038/331585a0

[bibr151-17470218241257885] Peterson L. R. Johnson S. T. (1971). Some effects of minimizing articulation on short-term retention. Journal of Verbal Learning and Verbal Behavior, 10, 346–354.

[bibr152-17470218241257885] Petrides M. Pandya D. N. (2009). Distinct parietal and temporal pathways to the homologues of Broca’s area in the monkey. PLOS Biology, 7(8), Article e1000170. 10.1371/journal.pbio.1000170 PMC2714989 19668354

[bibr153-17470218241257885] Price C. J. (2010). The anatomy of language: A review of 100 fMRI studies published in 2009. Annals of the New York Academy of Sciences, 1191, 62–88. 20392276 10.1111/j.1749-6632.2010.05444.x

[bibr154-17470218241257885] Price C. J. Moore C. J. Humphreys G. W. Wise R. J. (1997). Segregating semantic from phonological processes during reading. Journal of Cognitive Neuroscience, 9(6), 727–733. 23964595 10.1162/jocn.1997.9.6.727

[bibr155-17470218241257885] Raizada R. D. Poldrack R. A. (2007). Selective amplification of stimulus differences during categorical processing of speech. Neuron, 56(4), 726–740. 18031688 10.1016/j.neuron.2007.11.001

[bibr156-17470218241257885] Richardson J. T. E. Baddeley A. D. (1975). The effect of articulatory suppression in free recall. Journal of Verbal Learning and Verbal Behavior, 14, 623–629.

[bibr157-17470218241257885] Röer J. P. Bell R. Buchner A. (2015). Specific foreknowledge reduces auditory distraction by irrelevant speech. Journal of Experimental Psychology: Human Perception and Performance, 41, 692–702. 25621576 10.1037/xhp0000028

[bibr158-17470218241257885] Romani C. (1992). Are there distinct input and output buffers? Evidence from an aphasic patient with an impaired output buffer. Language and Cognitive Processes, 7(2), 131–162.

[bibr159-17470218241257885] Ruchkin D. S. Grafman J. Cameron K. Berndt R. S. (2003). Working memory retention systems: A state of activated long-term memory. Behavioral and Brain Sciences, 26(6), 709–728. 15377128 10.1017/s0140525x03000165

[bibr160-17470218241257885] Rushworth M. F. S. Behrens T. E. J. Johansen-Berg H. (2006). Connection patterns distinguish 3 regions of human parietal cortex. Cerebral Cortex, 16(10), 1418–1430. 16306320 10.1093/cercor/bhj079

[bibr161-17470218241257885] Rushworth M. F. S. Krams M. Passingham R. E. (2001). The attentional role of the left parietal cortex: The distinct lateralization and localization of motor attention in the human brain. Journal of Cognitive Neuroscience, 13(5), 698–710. 11506665 10.1162/089892901750363244

[bibr162-17470218241257885] Ryan J. (1969). Temporal grouping, rehearsal and short-term memory. Quarterly Journal of Experimental Psychology, 21, 148–155. 5787974 10.1080/14640746908400207

[bibr163-17470218241257885] Saffran E. M. Marin O. S. M. (1975). Immediate memory for word lists and sentences in a patient with deficient auditory short-term memory. Brain & Language, 2, 420–433. 1218376 10.1016/s0093-934x(75)80081-2

[bibr164-17470218241257885] Salamé P. Baddeley A. D. (1982). Disruption of short-term memory by unattended speech: Implications for the structure of working memory. Journal of Verbal Learning and Verbal Behavior, 21, 150–164.

[bibr165-17470218241257885] Salamé P. Baddeley A. D. (1986). Phonological factors in STM: Similarity and the unattended speech effect. Bulletin of the Psychonomic Society, 24, 263–265.

[bibr166-17470218241257885] Shallice T. Butterworth B. (1977). Short-term memory impairment and spontaneous speech. Neuropsychologica, 15, 729–735. 10.1016/0028-3932(77)90002-1 600367

[bibr167-17470218241257885] Shallice T. Papagno C. (2019). Impairments of auditory-verbal short-term memory: Do selective deficits of the input phonological buffer exist? Cortex, 112, 107–121. 30414628 10.1016/j.cortex.2018.10.004

[bibr168-17470218241257885] Shallice T. Warrington E. K. (1970). Independent functioning of verbal memory stores: A neuropsychological study. Quarterly Journal of Experimental Psychology, 22, 261–273. 5431401 10.1080/00335557043000203

[bibr169-17470218241257885] Shattuck-Hufnagel S. Klatt D. H. (1979). The limited use of distinctive features and markedness in speech production: Evidence from speech error data. Journal of Verbal Learning and Verbal Behavior, 18, 41–55.

[bibr170-17470218241257885] Sjöblom A. Hughes R. W. (2020). The articulatory determinants of verbal sequence learning. Journal of Experimental Psychology: Learning, Memory, and Cognition, 46(10), 1977–1997. 32700932 10.1037/xlm0000926

[bibr171-17470218241257885] Sternberg S. Wright C. E. Knoll R. L. Monsell S. (1980). Motor programs in rapid speech: Additional evidence. In Cole R. A. (Ed.), Perception and production of fluent speech (pp. 507–534). Erlbaum.

[bibr172-17470218241257885] Tremblay S. Macken W. J. Jones D. M. (2001). The impact of broadband noise on serial memory: Changes in band-pass frequency increase disruption. Memory, 9, 323–331. 10.1080/09658210143000010 11594355

[bibr173-17470218241257885] Vallar G. (2006). Memory systems: The case of phonological memory. A festschrift for Cognitive Neuropsychology. Cognitive Neuropsychology, 23, 135–155. 21049325 10.1080/02643290542000012

[bibr174-17470218241257885] Vallar G. Baddeley A. D. (1984). Fractionation of working memory: Neuropsychological evidence for a phonological short-term store. Journal of Verbal Learning and Verbal Behavior, 23, 151–161.

[bibr175-17470218241257885] Vallar G. Corno M. Basso A. (1992). Auditory and visual verbal short-term memory in aphasia. Cortex, 28(3), 383–389. 1395642 10.1016/s0010-9452(13)80148-7

[bibr176-17470218241257885] Vallar G. Di Betta A. M. Silveri M. C. (1997). The phonological short-term store-rehearsal system: Patterns of impairment and neural correlates. Neuropsychologica, 35, 795–812. 10.1016/s0028-3932(96)00127-3 9204486

[bibr177-17470218241257885] Vallar G. Papagno C. (2002). Neuropsychological impairments of verbal short-term memory. In Baddeley A. D. Wilson B. A. Watts F. N. (Eds.), Handbook of memory disorders (pp. 249–270). John Wiley.

[bibr178-17470218241257885] Vihman M. M. (2022). The developmental origins of phonological memory. Psychological Review, 129(6), 1495–1508. 35175093 10.1037/rev0000354

[bibr179-17470218241257885] Warren R. M. (1999). Auditory perception: A new analysis and synthesis. Cambridge University Press.

[bibr180-17470218241257885] Warren R. M. Obusek C. J. Farmer R. M. Warren R. P. (1969). Auditory sequence: Confusion of patterns other than speech or music. Science, 164, 586–587. 4888106 10.1126/science.164.3879.586

[bibr181-17470218241257885] Warrington E. K. Logue V. Pratt R. T. C. (1971). The anatomical localisation of selective impairment of auditory verbal short-term memory. Neuropsychologica, 9, 377–387. 10.1016/0028-3932(71)90002-9 5164373

[bibr182-17470218241257885] Warrington E. K. Shallice T. (1969). The selective impairment of auditory-verbal short-term memory. Brain, 92, 885–896. 5364015 10.1093/brain/92.4.885

[bibr183-17470218241257885] Warrington E. K. Shallice T. (1972). Neuropsychological evidence of visual storage in short-term memory tasks. Quarterly Journal of Experimental Psychology, 24, 423–431. 10.1080/14640747208400265 5017505

[bibr184-17470218241257885] Watkins M. J. Watkins O. C. Crowder R. G. (1974). The modality effect in free and serial recall as a function of phonological similarity. Journal of Verbal Learning and Verbal Behavior, 13(4), 430–447.

[bibr185-17470218241257885] Wickelgren W. A. (1964). Size of rehearsal group and short-term memory. Journal of Experimental Psychology, 68, 413–419. 14216501 10.1037/h0043584

[bibr186-17470218241257885] Wickelgren W. A. (1965). Short-term memory for phonemically similar lists. The American Journal of Psychology, 78(4), 567–574. 5839924

[bibr187-17470218241257885] Wickelgren W. A. (1966). Distinctive features and errors in short-term memory for English consonants. Journal of the Acoustical Society of America, 39, 388–398. 5907167 10.1121/1.1909900

[bibr188-17470218241257885] Wickelgren W. A. (1967). Rehearsal grouping and hierarchical organization of serial position cues in short-term memory. Quarterly Journal of Experimental Psychology, 19, 97–102. 6043059 10.1080/14640746708400077

[bibr189-17470218241257885] Wilding J. Mohindra N. (1980). Effects of subvocal suppression, articulating aloud and noise on sequence recall. British Journal of Psychology, 71, 247–261. 7378659 10.1111/j.2044-8295.1980.tb01742.x

[bibr190-17470218241257885] Woodward A. J. Macken W. J. Jones D. M. (2008). Linguistic familiarity in short-term memory: A role for (co-) articulatory fluency? Journal of Memory and Language, 58(1), 48–65.

[bibr191-17470218241257885] Zevin J. D. McCandliss B. D. (2005). Dishabituation of the BOLD response to speech sounds. Behavioral and Brain Functions, 1(1), 1–12. 15953396 10.1186/1744-9081-1-4 PMC1143777

